# Antibiotic Resistance: A Genetic and Physiological Perspective

**DOI:** 10.1002/mco2.70447

**Published:** 2025-10-31

**Authors:** Rania G. Elbaiomy, Ahmed H. El‐Sappah, Rong Guo, Xiaoling Luo, Shiyuan Deng, Meifang Du, Xiaohong Jian, Mohammed Bakeer, Zaixin Li, Zhi Zhang

**Affiliations:** ^1^ Department of Biological Engineering Sichuan University of Science & Engineering Zigong China; ^2^ College of Agriculture Forestry, and Food Engineering Yibin University Yibin Sichuan China; ^3^ Department of Genetics Faculty of Agriculture Zagazig University Zagazig Egypt; ^4^ Department of Gastroenterology FuShun People's Hospital Zigong China; ^5^ Division of Hematology and Medical Oncology Mayo Clinic Jacksonville Florida USA; ^6^ Division of Internal Medicine‐Clinical Hematology Al‐Azhar University Cairo Egypt

**Keywords:** β‐lactamases, antibiotic resistance, biofilm, coccoid, efflux pumps, genetic mutations

## Abstract

Antimicrobial‐resistant bacteria, a growing worldwide concern, reduce the effectiveness of antibiotics against a wide range of microbial infections. Various bacterial species have quickly developed antibiotic resistance since the first mention of penicillin resistance in 1947. A rise in mortality, more extended hospital stays, more healthcare expenditures, and morbidity are all brought about by these bacteria that are resistant to antibiotics. To develop resistance, bacteria may undergo genetic changes, engage in horizontal gene transfer, produce β‐lactamase, activate efflux pumps, form biofilms, and alter their metabolism to become less susceptible to drugs. Environmental factors and sublethal antibiotic exposure exacerbate resistance, particularly in cases of persistent infections caused by biofilms. This tendency is prompted by the overuse of antibiotics in both human and veterinary medicine, as well as inadequate infection control measures and environmental pollution. This review presents an extensive survey of antimicrobial resistance across bacterial taxa, with a focus on the physiological and genetic processes underlying this phenomenon. It delves into the current therapeutic landscape and showcases cutting‐edge methods—such as artificial intelligence‐driven antibiotic discovery and resistance prediction—to inform the development of next‐generation antibiotics and containment systems.

## Graphical abstract

The development of resistance to antimicrobials and their historical progression are depicted in this graphic. It draws attention to important biochemical, physiological, and genetic factors that contribute to AMR, such as the transmission of genes, the development of biofilms, and the inactivation of antibiotics. Treatment milestones are shown in relation with the growth of resistance on the timeline. To combat the growing problem of antibiotic resistance, new treatment options are showing promise, such as host‐directed treatments and AI‐guided treatments creation.

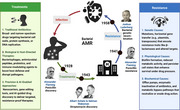

## 1 Introduction

Bacterial antimicrobial resistance (AMR) is a global health concern that has accelerated in recent decades, primarily due to the widespread use of antibiotics during the 20th century [1, 2]. Antibiotics, once hailed as “miracle drugs,” revolutionized medicine by enabling the treatment of infectious diseases caused by a wide range of harmful microorganisms [3]. However, the excessive and sometimes improper use of these drugs in human healthcare, veterinary care, and agriculture has accelerated the emergence and spread of antibiotic‐resistant bacteria [4]. AMR has become a serious public health problem worldwide, as its prevalence spreads across ecosystems and continents [5]. The World Health Organization (WHO) has consistently ranked antibiotic resistance as one of the top 10 global health threats due to its significant impact on clinical outcomes and economic stability [6, 7].

Prolonged hospital admissions, increased healthcare costs, and higher mortality rates have been attributed to the emergence of resistance across a broad spectrum of microbial diseases, including fungi, mycobacteria, and both Gram‐negative and Gram‐positive bacteria. Infections caused by multidrug‐resistant (MDR) organisms, including *Escherichia coli* (*E. coli*), *Klebsiella pneumoniae* (*K. pneumoniae*), *Pseudomonas aeruginosa* (*P. aeruginosa*), *Acinetobacter baumannii* (*A. baumannii*), *Staphylococcus aureus* (*S. aureus*), *Enterococcus faecium* (*E. faecium*), and *Mycobacterium tuberculosis* (*M. tuberculosis*), are becoming increasingly severe and difficult to treat [8]. These microorganisms employ various resistance strategies, including enzymatic antibiotic degradation, target‐site modification, efflux pump activation, biofilm formation, reduced membrane permeability, and metabolic reprogramming, among others [9, 10, 11].

In addition to more established genetic factors like *mecA* and *vanA*, new mutations continue to emerge in Gram‐positive pathogens, rendering them less susceptible to last‐line antibiotics, such as daptomycin and linezolid [12]. Because of their unique cell envelope—a thin peptidoglycan layer protected by an outer membrane rich in lipopolysaccharides (LPS)—Gram‐negative bacteria have inherent resistance [10, 13]. The presence of several efflux pumps amplifies their capacity to evade antimicrobial activity, and this outer membrane serves as an impenetrable barrier to many antibiotics [14]. Resistance in Gram‐positive bacteria typically develops through horizontal gene transfer (HGT) or changes to penicillin‐binding proteins (PBPs). Antibiotic resistance in mycobacteria is achieved through gene mutations and the regulation of efflux activity. Changes in ergosterol production, alterations in drug target genes, and upregulation of efflux transporters are some mechanisms by which fungal infections, such as those caused by *Aspergillus fumigatus* and *Candida albicans*, exhibit resistance [11].

The HGT, which encompasses conjugation, transformation, and transduction, facilitates the rapid acquisition and transmission of resistance genes via plasmids, integrons, and transposons [15]. These elements often include clusters of resistance genes, allowing bacteria such as *E. coli*, *K. pneumoniae*, and *S. aureus* to tolerate multiple antibiotics simultaneously [16]. Collateral resistance and sensitivity networks, in which hypersensitivity to one therapy results in resistance to another, are becoming increasingly essential in the development of AMR. New pharmaceutical combinations that leverage these interactions through chemical genetic profiles offer a novel approach to combating antibiotic resistance. Soil, water, animals, and agricultural systems are increasingly infested with resistant microbes and resistance genes, resulting in a global resistome that promotes the transfer of resistance factors between environmental and clinical strains [3, 17], emphasizing the ecological component of AMR. Sublethal antibiotic doses are one environmental stressor that causes the development of biofilm‐forming phenotypes and resistance variants. Furthermore, bacterial and fungal infections may evade treatments that primarily target actively growing cells by adopting low‐metabolic or persistent states [11, 18].

To combat the growing issue of antibiotic resistance in Gram‐positive bacteria, scientists have developed multiarmed chemical scaffolds to aid in the creation of novel medicines. These are multiarmed antibiotics (MAAs), comprised of multiarmed molecules with an inert core and several inactive arms. MAAs with cores such as benzene, ethylene, carbon, nitrogen, or triazine and arms such as phenylbenzoic acid, vinylbenzoic acid, or ethynylbenzoic acid may effectively reduce Gram‐positive bacteria by targeting the lipid carriers involved in cell wall production. Based on their excellent findings against clinical MDR bacteria, these compounds offer promising possibilities for developing novel antibiotics [19]. Furthermore, molecular and computational technologies such as next‐generation sequencing (NGS), metagenomics, clustered regularly interspaced short palindromic repeats–CRISPR‐associated proteins (CRISPR–Cas systems), and artificial intelligence (AI) are becoming increasingly crucial in current efforts to combat AMR. These approaches may uncover novel resistance genes, predict how resistance will evolve, and aid in the development of new treatments [16, 20]. This illustrates a potential pathway for drug innovation. These advances are crucial for identifying novel resistance genes, understanding their regulation, and developing future antimicrobials. However, research on antibiotic treatments is lagging behind the pace of resistance evolution, underscoring the crucial need for increased international collaboration, enhanced monitoring, and novel treatment options.

This review aims to provide a comprehensive understanding of AMR in all major bacterial pathogens. It examines the genetic and physiological causes, environmental factors, antibiotic‐based strategies that decrease resistance, and novel techniques to combat this significant public health issue.

## 2 The Evolution of Antibiotic Resistance: A Historical Perspective

Antibiotic resistance has hindered the treatment of infectious diseases since the early days of modern antibiotics [21]. Antibiotic resistance has been a long‐standing issue, but recent attention has brought it to the forefront as never before. The ongoing evolutionary arms race between bacteria and their competitors, encompassing both naturally occurring and synthetic substances, is the root cause of this issue [22]. Since the advent of antimicrobial treatments, the rapid evolution of resistance mechanisms has been a recurring issue affecting the efficacy of antibiotics in treating diseases [22]. The introduction of sulfonamides in the 1930s marked the onset of antibiotic resistance [16, 23]. These were the pioneering medications in chemotherapy. Nonetheless, bacteria soon developed resistance, which is now a major issue, particularly with Gram‐negative bacteria, posing a significant threat to global public health. This historical trend of resistance is inseparably linked to the unique action mechanisms of antimicrobial drugs. An effective mechanism of action for each antibiotic class is to selectively target a specific, often unique, bacterial structure or pathway; this process generates intense natural selection pressure, which in turn leads to the evolution of resistance [24]. The complex process of cell wall construction is targeted by the β‐lactam family of antibiotics, which includes carbapenems, cephalosporins, penicillins, and glycopeptides such as vancomycin [25]. To induce cell lysis, these antibiotics bind to PBPs or the d‐Ala–d‐Ala termini of peptide precursors [25]. Polymyxins, such as colistin, disrupt the LPS layer of Gram‐negative bacteria, allowing them to target the outer membrane. Aminoglycosides (e.g., gentamicin) and macrolides (e.g., erythromycin) bind to the 30S ribosomal subunit to induce mistranslation, and oxazolidinones and lincosamides attach to the 50S subunit to prevent peptide elongation [26]. This class of inhibitors has a broad impact on protein synthesis.

Another subunit that tetracyclines aim to block is the 30S subunit, which is involved in the attachment of tRNA. Some types aim to prevent nucleic acid production; for example, rifamycins inhibit RNA polymerase, while fluoroquinolones (such as ciprofloxacin) inhibit DNA gyrase and topoisomerase IV [27, 28]. Another weakness is the metabolic pathways sequentially blocked during folate synthesis by sulfonamides and trimethoprim. Bedaquiline, which targets ATP synthase directly, and isoniazid, which blocks mycolic acid synthesis, are two medications used to treat mycobacterial infections [29]. The β‐glucan cell wall and fungal‐specific components, such as ergosterol, are targeted by antifungal drugs like azoles, echinocandins, and polyenes. Selective antimicrobial toxicity is based on the specificity of these cellular targets, which also defines the battlefield where resistance develops [30]. Bacteria have developed resistance mechanisms for every targeted pathway, resulting in the complex resistance landscape discussed later. These mechanisms include target modification, drug inactivation, and reduced permeability.

The concept of selective antimicrobial toxicity was first proposed by Paul Ehrlich in 1909, following the development of Salvarsan (arsphenamine), a chemical based on arsenic that was used to treat syphilis and trypanosomal infections [31]. Chemotherapy reached a new peak with the development of Salvarsan, one of the earliest synthetic antibacterial agents. Salvarsan took more than 20 years to develop resistance to, in contrast to sulfonamides and penicillin, which both faced resistance within about 12 years. Throughout the 20th century, it was widely accepted that Ehrlich's prediction that diseases would adapt and propagate resistance traits over generations was correct. The pivotal event began in September 1928, when Alexander Fleming inadvertently discovered penicillin [32, 33]. In 1940, clinical approval was granted, which coincided with a significant discovery by Edward Abraham and Ernst Chain. These scientists discovered an enzyme in *E. coli* called β‐lactamase, which could hydrolyze penicillin. This discovery revealed a mechanism of resistance that had already been present [32, 33]. The first human to receive penicillin was police officer Albert Alexander, who was tested for the antibiotic's medicinal potential in 1941.

There were initial indications of improvement, but the treatment was unsuccessful due to insufficient medication [34, 35]. To combat this, Florey and colleagues developed innovative ways to purify urine of penicillin, concentrate it, and then recycle it, allowing therapeutic concentrations to be maintained [36]. Although penicillin ushered in a new era of treatment for infectious diseases, its widespread use led to bacteria undergoing intense natural selection. Clinical underdosing can potentially encourage the development of resistant strains in patients, as Fleming cautioned in his 1945 Nobel Lecture. Resistance can be readily produced in the laboratory using sublethal doses [34, 35]. A pandemic of antibiotic resistance was marked by the emergence of penicillin‐resistant *S. aureus* in 1947, one of the first recorded instances of such a hazard in patients’ health. Gram‐negative bacteria were the first to evolve resistance, while Gram‐positive species followed suit, often due to chromosomal changes or the introduction of mobile genetic elements [32]. The rapid spread of Gram‐negative bacteria resistant to several drugs quickly became a serious public health concern, and this adaptive surge was most noticeable in healthcare facilities. The emergence of β‐lactamase‐producing *E. coli* strains and other intestinal Gram‐negative bacteria illustrates the rapid development of resistance to penicillin and subsequent generations of antibiotics [34, 35]. The outer membrane of these bacteria rendered them more formidable, functioning as a formidable barrier that impedes the penetration of antibiotics, thereby increasing resistance [37, 38].

The decades after World War II marked the beginning of a golden era of antibiotic development. We witnessed the rapid discovery and implementation of new antibiotic families, including aminoglycosides, tetracyclines, cephalosporins, macrolides, and fluoroquinolones. However, resistance continued to grow. One of the first successful tuberculosis (TB) therapies, streptomycin, was quickly rendered ineffective due to alterations at the target sites (rpsL and rrs) [39, 40]. Isoniazid was introduced in the 1950s and promptly developed resistance when taken alone, necessitating combination treatment. Gram‐negative bacteria have rapidly developed resistance due to chromosomal alterations and enzyme inactivation [37, 41]. The discovery of plasmid‐mediated aminoglycoside resistance was a watershed moment in the history of resistance, establishing HGT as the primary cause [4, 42].

In the 1950s, HGT was established as the primary resistance mechanism in plasmid‐mediated aminoglycoside resistance. Scientists have made a significant discovery: *Shigella* and *E. coli* can transmit conjugative plasmids [15, 43]. This study found evidence that certain bacteria, known as Gram‐negative, could transmit resistance genes from one species to another. Investigative advancements showed gene transfer between *Shigella* and *E. coli*, revealing the transmission of genes between species. Microbes swiftly develop antibiotic resistance after being conjugated, converted, or transduced by a phage. This discovery altered our understanding of how AMR spreads in nosocomial infections. Gram‐negative bacteria, such as *K. pneumoniae, P. aeruginosa*, and *A. baumannii*, rapidly develop resistance to many pharmaceuticals [8, 37, 44].

By the 1970s and 1980s, Enterobacteriaceae had produced extended‐spectrum β‐lactamases (ESBLs), such as CTX‐M, SHV, and TEM, which weakened the effectiveness of third‐generation cephalosporins [34, 45]. Bacterial antibiotic resistance may be linked to carbapenemases, porin loss (e.g., OmpK35/36 in *K*. *pneumoniae*), and efflux pump overexpression (e.g., MexAB–OprM in *P. aeruginosa*) [40, 46, 47]. When these systems interact, antibiotic‐based strategies become ineffective. Extra barriers created by the outer membranes of Gram‐negative bacteria were often observed in association with porin loss and overexpression of efflux pumps. These findings demonstrate how Gram‐negative bacteria are highly adaptable, enabling them to develop resistance through multiple routes simultaneously. The evolution of Gram‐positive bacteria happened concurrently. While methicillin‐resistant *S. aureus* (MRSA) emerged when *S. aureus* acquired the *mecA* gene, *E. faecium* developed vancomycin resistance via the *vanA* and *vanB* genes [37, 38]. This is because Gram‐positive bacteria lack an outer membrane and complex HGT mechanisms, unlike Gram‐negative bacteria [13, 38].

The proliferation of mobile genetic elements, such as transposons, integrons, and integrative conjugative elements (ICEs), has accelerated the historical development of resistance in Gram‐negative bacteria [43, 48]. These elements facilitate the cotransmission of resistance and virulence genes. The environmental and commensal reservoirs of resistance genes have exacerbated this process by serving as additional sources of antibiotic resistance transmission [4, 17]. Resistance has transcended hospitals and clinics, complicating the struggle against AMR. Antibiotics are widely used in agriculture, especially in the United States. To promote growth and prevent diseases, cattle were fed subtherapeutic doses. This strategy created reservoirs of resistance genes. More than half of the antimicrobials sold to the agriculture sector were classified as “medically important” by 2021. Clinical doses were linked to agricultural AMR via vectors such as soil, wastewater, and food chains. In the early 1990s, the need for pharmaceutical marketing also increased. Salespeople often exaggerated the benefits of antibiotics. “My new third‐generation cephalosporin is the drug if you don't know the bug,” a sales representative claimed. Even though antibiotics are ineffective against viruses, another study promoted that fluoroquinolones should be used throughout the cold and flu season. These efforts worsened resistance and overprescribing.

The emergence of MDR ESKAPE diseases, including *K. pneumoniae*, *A. baumannii*, and *P. aeruginosa*, underscores the need to address Gram‐negative bacteria in the fight against antibiotic resistance [49]. The global burden of antibiotic‐resistant diseases and mortality is increasing, and intractable infections are a key contributor. The development of hypervirulent, carbapenem‐resistant *K. pneumoniae* underscores the intricate interplay between resistance and virulence, making treatment decisions and clinical outcomes more challenging [50, 51]. Vaccination expansion, diagnostic improvement, reduction of antibiotic exposure to nonhumans through One Health, stewardship optimization, and continued investment in novel antibiotic pipelines have all been recognized as major priorities.

**FIGURE 1:**
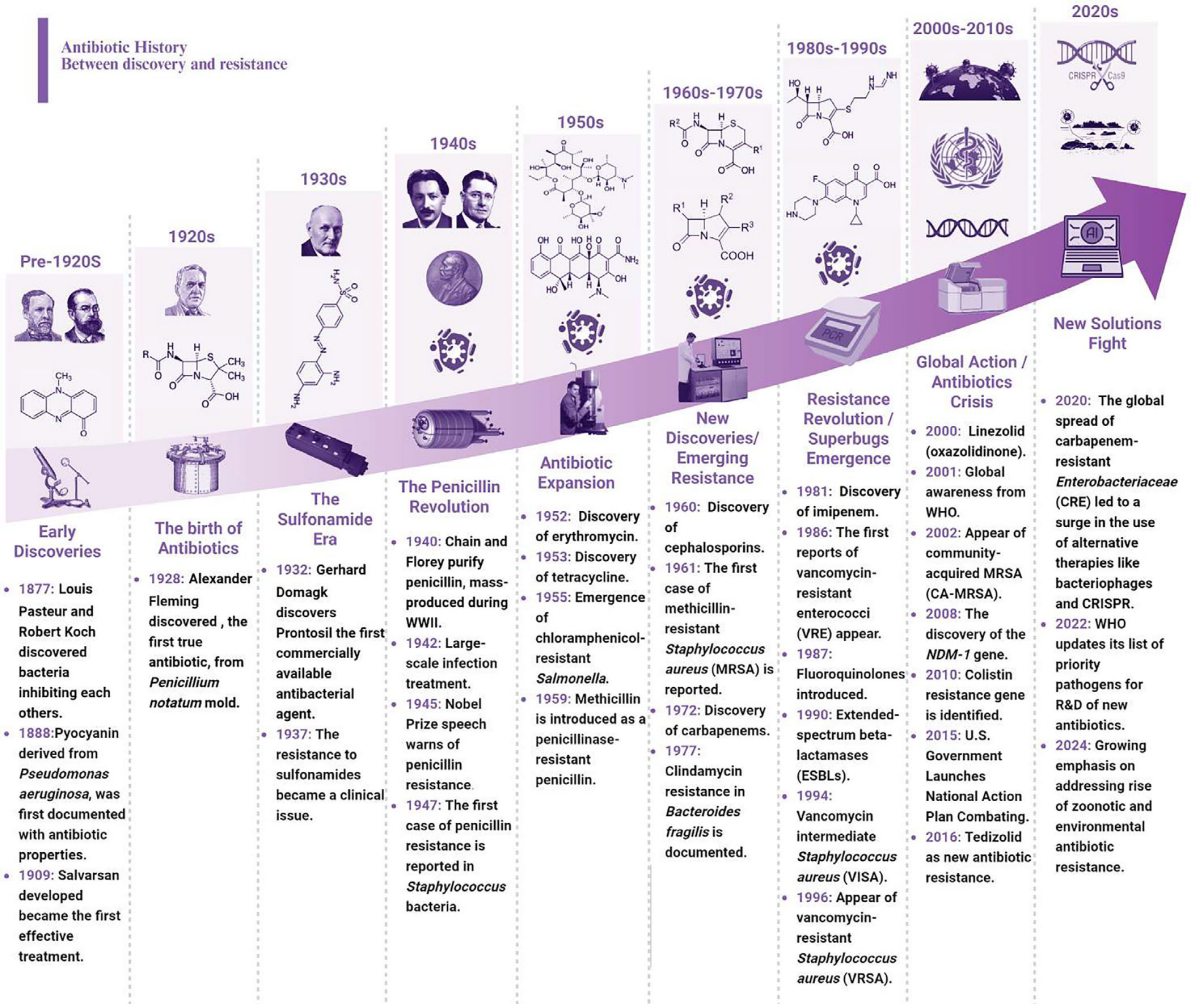
The brief history of antibiotic discoveries and resistance. The raised arrow highlights significant improvements in equipment that aided scientists in accomplishing their study objectives. This set of tools includes a light microscope, an autoclave (an early form of pressure steriliser), a centrifuge rotor (an early form of ultracentrifuge component), a transmission electron microscope (TEM), an early protein sequencer, a polymerase chain reaction (PCR) machine, a modern automated DNA sequencer, and a computer with artificial intelligence capabilities. Figure created using BioRender.

Ultimately, due to its spread mechanisms, antibiotic resistance affects all types of bacteria, particularly those of the Gram‐negative variety. Their wide range of potent resistance mechanisms and capacity to rapidly acquire and transmit genetic material make them an essential target for global health monitoring and intervention programs [52, 53]. Antibiotic resistance is becoming a more severe issue for global health, as seen by the emergence of the problem in Gram‐negative bacteria. The discovery of novel medications, advancements in diagnostic techniques, and growing international cooperation are all crucial in combating antibiotic resistance, particularly against Gram‐negative bacteria. The concise history of antibiotic discoveries and resistance, illustrated in Figure 1, outlines the timeline of antibiotic development, marked by the simultaneous rise of resistance. This provides insight into the ongoing battle against antibiotic resistance.

## 3 Fundamental Mechanisms Driving Drug Resistance

**FIGURE 2:**
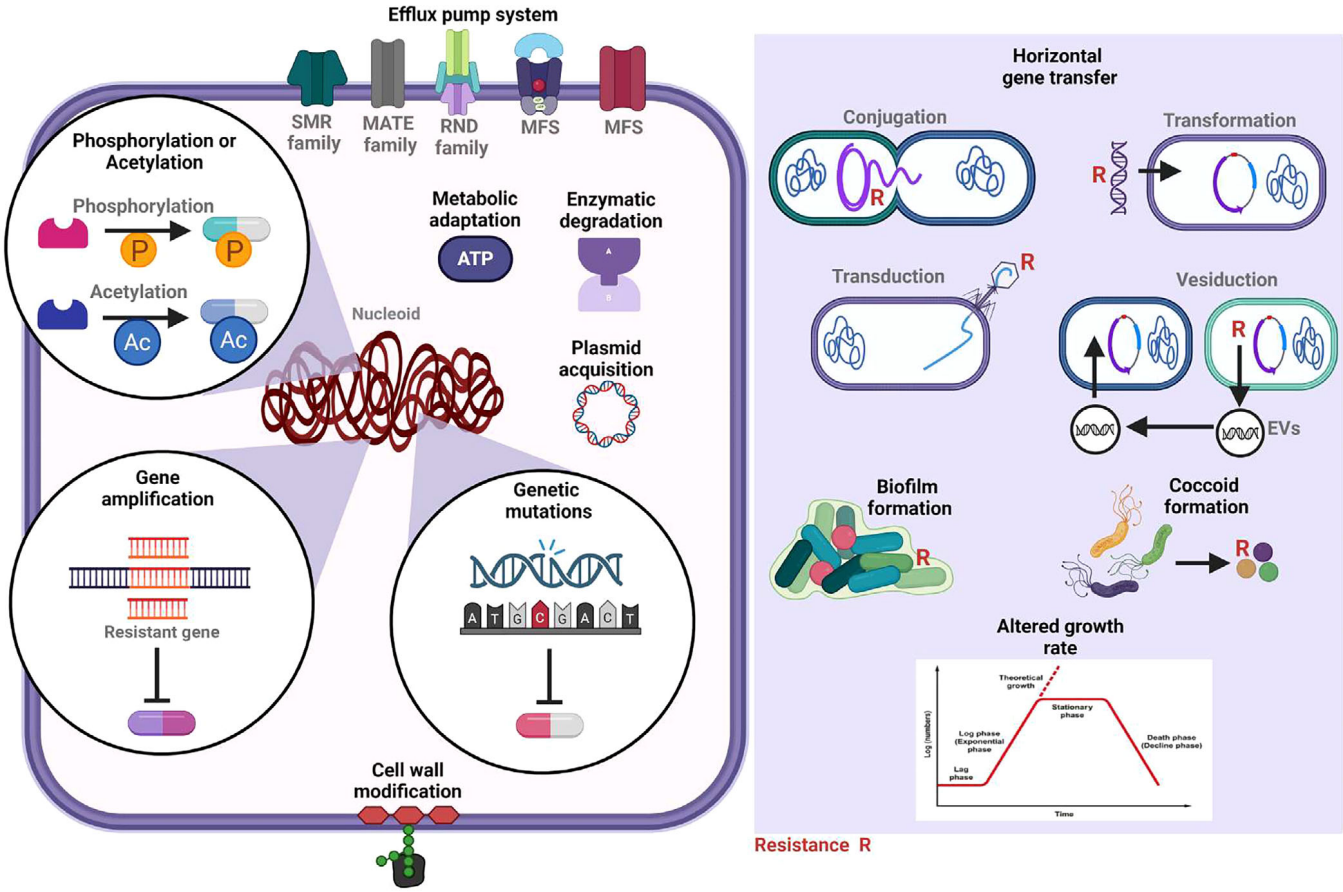
Mechanisms by which bacteria develop resistance to antibiotics. Bacteria utilize efflux pump systems, including MATE, ABC, MFS, and SMR, to actively remove antimicrobial compounds from their cells, thereby reducing the concentration and efficacy of these compounds. Many bacterial species, such as mycobacteria and Gram‐positive pathogens, possess similar transporters; a notable example is the MexAB–OprM efflux system. The fast development of multidrug resistance is aided by the dissemination of resistance genes throughout bacterial populations through horizontal gene transfer and plasmid acquisition. Enzymatic degradation of β‐lactam antibiotics, such as penicillins and carbapenems, can be facilitated by β‐lactamases, which many bacteria produce. These include ESBLs and carbapenemases. The structural alteration of antibiotics by enzymes such as acetyltransferases and phosphotransferases, which hinders drug function, is another essential resistance mechanism. Gene alterations that affect antibiotic target sites, including ribosomal proteins, RNA polymerase, or DNA gyrase, can also diminish the binding and effectiveness of the medicine. Furthermore, bacteria can modify target molecules, rendering antibiotics ineffective through chemical modification. In particular, persistent infections benefit from the sluggish growth rates and hibernation of biofilms, which physiologically contribute to increased resistance. Biofilms form a protective matrix that prevents cells from being attacked by the immune system and limits the penetration of antibiotics. Further alterations to the bacterial cell wall, such as changes to membrane proteins or porins, can further restrict the entrance of antibiotics. Antimicrobial resistance is on the rise worldwide among various types of microbes, and the multiple processes involved, including efflux, enzymatic inactivation, genetic mutation, and physiological adaptation, make it very challenging to treat bacterial infections. Figure created using BioRender.

Antibiotic resistance may be caused by various factors, including human‐related, environmental, physiological, biochemical, and genetic factors. One human variable that may contribute to bacteria acquiring tolerance to subsequent antibiotic treatment is the unethical use of antibiotics, especially in areas with limited medical knowledge [54]. Various causes of antibiotic resistance in bacteria have been examined, including genetic, physiological, and metabolic alterations (Figure 2 and Table 1) [55, 56, 57].

**TABLE 1:** Antibiotic resistance mechanisms in bacteria.

Bacterial species	Antibiotic(s)	Types of resistance	Cause of resistance	References
Gram‐negative bacteria *H. pylori*	Metronidazole, rifampin, tetracyclines, fluoroquinolones, amoxicillin, levofloxacin, and clarithromycin	Nitroreductase mutations, efflux pumps, DNA gyrase mutations, penicillin‐binding protein mutations, *rpoB* mutations, and point mutations in ribosomal RNA	Nitroreductase mutations (metronidazole resistance), efflux pumps (multiple medicines resistance), *rpoB* gene mutations (rifampin resistance), *gyrA* mutations (fluoroquinolones and levofloxacin resistance), *pbp1A* mutations (amoxicillin resistance), and point mutations in *23S rRNA* (clarithromycin resistance)	[58, 59, 60, 61, 62, 63]
*E. coli*	Fluoroquinolones, β‐lactams, aminoglycosides, polymyxins, tetracyclines, sulfonamides, macrolides, chloramphenicol, and carbapenems	Efflux pumps produce β‐lactamase, ribosomal target mutations, *MCR* gene, porin loss, acetyltransferases, tetracycline efflux pumps, dihydropteroate synthase mutations, and carbapenemase production.	Mutations in *gyrA* and *parC* (fluoroquinolones resistance), extended‐spectrum beta‐lactamases (ESBL) production (β‐lactams resistance), acquisition of *mcr* genes (polymyxins resistance), loss of porins (carbapenems resistance), aminoglycoside‐modifying enzymes, efflux pumps (tetracyclines, macrolides resistance), acetyltransferase production (chloramphenicol resistance), and mutations in dihydropteroate synthase (sulfonamides resistance) are all potential mechanisms.	[64, 65, 66, 67, 68, 69, 70, 71]
*K. pneumoniae*	Carbapenems, β‐lactams, colistin, fluoroquinolones, aminoglycosides, tigecycline, cephalosporins, tetracyclines, and fosfomycin	Carbapenemae production (KPC, NDM, OXA), ESBL production, efflux pumps, and target site mutations, the absence of the porin, *TetA*/B, *fosA*, and *mcr* genes, and plasmid‐mediated resistance	KPC/NDM carbapenemase (carbapenems resistance), ESBL production (β‐lactams, cephalosporins resistance), *mcr* gene (colistin resistance), mutations in *gyrA* and *parC* (fluoroquinolones resistance), porin loss (carbapenems resistance), aminoglycoside‐modifying enzymes, *TetA*/B efflux pumps (tetracyclines resistance), and *fosA* gene (fosfomycin resistance) are all potential therapeutic targets.	[72, 73, 74, 75, 76, 77, 78, 79]
*N. gonorrhoeae*	Penicillin, tetracyclines, macrolides, fluoroquinolones, cephalosporins, aminoglycosides, sulfonamides, azithromycin, and ceftriaxone	Penicillinase production, altered penicillin‐binding proteins (PBPs), efflux pumps, target site mutations, plasmid‐mediated resistance, ribosomal protection, *TetM* gene, *porB* mutations, *gyrA*, *parC* mutations, and mosaic *penA* alleles	Penicillinase (penicillin resistance), *TetM*‐mediated efflux (tetracycline resistance), ribosomal protection proteins (macrolide resistance), mutations in *gyrA*/*parC* (fluoroquinolones resistance), mosaic *penA* alleles (cephalosporins resistance), mutations in 16S rRNA (aminoglycoside resistance), mutations in dihydropteroate synthase (sulfonamides resistance), altered *PBPs* (β‐lactams, cephalosporins resistance)	[80, 81, 82, 83, 84, 85, 86, 87]
*A. baumannii*	Carbapenems, colistin, fluoroquinolones, aminoglycosides, β‐lactams, cephalosporins, tetracyclines, polymyxins, sulfonamides, tigecycline, rifampin, chloramphenicol, and trimethoprim‐sulfamethoxazole	Carbapenemase production (OXA‐23, OXA‐24, OXA‐51, NDM, IMP), efflux pumps (AdeABC, AdeIJK, AdeFGH, RND), porin loss (CarO), mcr genes, target site mutations (gyrA, parC), TetA/B genes, altered PBP, AAC(6′)‐Ib, rmt genes, mutations in LPS synthesis, sul genes, and plasmid‐mediated resistance	OXA‐23, OXA‐24, OXA‐51, NDM, IMP (carbapenem resistance), mcr‐1 and mcr‐2 (colistin resistance), AdeABC, AdeIJK (multidrug efflux), *gyrA* and *parC* mutations (fluoroquinolone resistance), resistance/B genes (tetracycline resistance), *sul1*/*sul2* (sulfonamide resistance), porin loss (CarO for β‐lactams), AAC(6')‐Ib (aminoglycosides), and mutations in LPS synthesis (polymyxin resistance)	[88, 89, 90, 91, 92, 93, 94, 95, 96]
*P. aeruginosa*	Carbapenems, β‐lactams, fluoroquinolones, aminoglycosides, polymyxins, tetracyclines, cephalosporins, monobactams, sulfonamides, rifampin, chloramphenicol, fosfomycin, and macrolides	Carbapenemase production (IMP, VIM, NDM, OXA), efflux pumps (MexAB–OprM, MexXY–OprM, MexCD–OprJ, MexEF–OprN), porin loss (OprD), target site mutations, RND pumps, AAC(6')‐Ib, *rmt* genes, *MCR‐1*, *GyrA*/*ParC* mutations, plasmid‐mediated resistance, d‐alanyl–d‐alanine modification, and LPS modification	IMP, VIM, NDM, OXA carbapenemases (carbapenem resistance), MexAB–OprM (β‐lactam and fluoroquinolone resistance), *GyrA*/*ParC* mutations (fluoroquinolone resistance), MexXY–OprM (aminoglycoside resistance), AAC(6')‐Ib, *rmt* genes (aminoglycoside resistance), MCR‐1 (polymyxin resistance), *OprD* porin loss (β‐lactams and carbapenems resistance), d‐alanyl–d‐alanine modifications (vancomycin resistance), and LPS modification (colistin resistance)	[97, 98, 99, 100, 101, 102, 103, 104, 105]
*S. enterica*	Ampicillin, trimethoprim‐sulfamethoxazole, tetracyclines, fluoroquinolones, ciprofloxacin, azithromycin, chloramphenicol, ceftriaxone, gentamicin, and carbapenems	The ESBLs, AmpC β‐lactamase, chromosomal mutations, efflux pumps (AcrAB–TolC), target site mutations (*gyrA*, *parC*), plasmid‐mediated resistance (blaCTX‐M, *blaTEM*), *rmtA*, methylation of rRNA, *TetA*/B, *Sul1*/*Sul2*, and *Van* genes	ESBLs (ampicillin resistance), AmpC β‐lactamase (cephalosporin resistance), *GyrA* and *ParC* mutations (fluoroquinolones resistance), AcrAB–TolC efflux system (multidrug resistance), plasmid‐mediated resistance (blaCTX‐M, blaTEM), *rmtA* (aminoglycoside resistance), methylation of rRNA (macrolides resistance), *TetA*/B (tetracyclines resistance), and *Sul1*/*Sul2* (sulfonamides resistance)	[106, 107, 108, 109, 110, 111, 112, 113, 114]
*H. influenzae*	Ampicillin, ceftriaxone, azithromycin, chloramphenicol, tetracyclines, trimethoprim–sulfamethoxazole, and rifampin	β‐Lactamase production (TEM, SHV, CTX‐M), altered PBPs, efflux pumps, mutations in ribosomal RNA (rRNA), plasmid‐mediated resistance, the *ermB* gene, *TetM*, *Sul1*/*Sul2*, and *gyrA* mutations	β‐Lactamases (ampicillin and cephalosporin resistance), altered PBPs (cephalosporin resistance), efflux pumps (multidrug resistance), rRNA mutations (macrolide resistance), *ermB* (macrolide resistance), *TetM* (tetracycline resistance), *Sul1*/*Sul2* (sulfonamide resistance), and *gyrA* mutations (fluoroquinolone resistance)	[115, 116, 117, 118, 119, 120, 121]
*E. cloacae*	Ampicillin, cephalosporins, carbapenems, fluoroquinolones, trimethoprim–sulfamethoxazole, tetracyclines, and aztreonam	The ESBLs, carbapenemases (KPC, NDM), AmpC β‐lactamase, efflux pumps (AcrAB), mutations in PBPs, plasmid‐mediated resistance, methylation of rRNA, *qnr*, *Sul1*/*Sul2* genes, and *TetA* gene	ESBLs (ampicillin and cephalosporin resistance), carbapenemases (carbapenem resistance), AmpC β‐lactamase (β‐lactam resistance), AcrAB efflux pumps (multidrug resistance), mutations in PBPs (cephalosporin), rRNA methylation (macrolides resistance), *qnr* gene (fluoroquinolone resistance), and *TetA* (tetracycline resistance)	[122, 123, 124, 125, 126, 127, 128, 129]
*Shigella spp*.	Ampicillin, trimethoprim–sulfamethoxazole, fluoroquinolones, azithromycin, ceftriaxone, tetracyclines, and gentamicin	The ESBLs, plasmid‐mediated AmpC β‐lactamase, efflux pumps (AcrAB), mutations in target sites (*gyrA*, *parC*), methylation of rRNA, *qnr* genes, *Sul1*/*Sul2* genes, and tetracycline resistance genes (*tetA*, and *tetM*)	ESBLs (ampicillin resistance), plasmid‐mediated AmpC β‐lactamase (cephalosporin resistance), AcrAB efflux pumps (multidrug resistance), mutations in *gyrA* and *parC* (fluoroquinolone resistance), rRNA methylation (macrolide), *qnr* genes (fluoroquinolone resistance), *Sul1*/*Sul2* (sulfonamide resistance), tetA and tetM (tetracycline resistance)	[130, 131, 132, 133, 134, 135, 136, 137]
*C. jejuni*	Macrolides, fluoroquinolones, tetracyclines, ampicillin, gentamicin, chloramphenicol, and trimethoprim–sulfamethoxazole	Efflux pumps (CmeABC), mutations in *gyrA* and *parC*, A2074G mutation in 23S rRNA, plasmid‐mediated resistance, tetracycline resistance genes (tetO, tetM), aminoglycoside‐modifying enzymes (aac(3)‐IV), and carbapenemase production (rare)	Efflux pumps (multidrug resistance), plasmid‐mediated resistance, rare carbapenemases (carbapenem resistance), mutations in *gyrA* and *parC* (fluoroquinolone resistance), A2074G mutation in 23S rRNA (macrolide resistance), *tetO* and *tetM* (tetracycline resistance), and aac(3)‐IV (gentamicin resistance)	[138, 139, 140, 141, 142, 143, 144]
Gram‐positive bacteria *S. aureus*	Penicillin, methicillin, vancomycin, linezolid, tetracyclines, daptomycin, aminoglycosides, macrolides, and fluoroquinolones	β‐Lactamase production, altered PBPs, cell wall thickening, target site mutation, ribosomal protection proteins, membrane charge alteration, aminoglycoside‐modifying enzymes, efflux pumps, and DNA gyrase mutations.	Acquisition of *β‐lactamase* genes (penicillin resistance), *mecA* gene (methicillin resistance), thickened cell wall (vancomycin resistance), mutation in *23S rRNA* gene (linezolid resistance), *tetM* gene (tetracycline resistance), altered membrane charge (daptomycin resistance), aminoglycoside‐modifying enzymes, efflux pumps (macrolide resistance), mutations in *gyrA* and *parC* (fluoroquinolone resistance)	[47, 48, 49, 50, 51, 52, 53, 54]
*E. faecium*	Vancomycin, linezolid, daptomycin, aminoglycosides, tetracyclines, β‐lactams, quinupristin–dalfopristin, macrolides, and chloramphenicol	*VanA*/*VanB* genes, rRNA mutations, LiaFSR mutations, efflux pumps, *TetM*/*TetL* genes, altered PBPs, ErmB methylation, AAC(6′)‐Ie‐APH(2′′) enzyme, plasmid‐mediated resistance, and multidrug efflux pumps	*VanA*/*VanB* genes (vancomycin resistance), 23S rRNA mutations (linezolid resistance), LiaFSR mutations (daptomycin resistance), TetM/TetL (tetracycline resistance), altered PBPs (β‐lactams), ErmB methylation (macrolides resistance), AAC(6′)‐Ie‐APH(2′′) enzyme (aminoglycosides resistance), plasmid‐mediated resistance (chloramphenicol resistance), multigene efflux systems (multiple antibiotics resistance)	[89, 90, 91, 92, 93, 94, 95, 96, 97]
*S. pneumoniae*	Penicillin, macrolides, tetracyclines, trimethoprim–sulfamethoxazole, vancomycin, ceftriaxone, chloramphenicol, and rifampin	Penicillin‐binding protein (PBP) mutations, methylation of rRNA, efflux pumps (*mefE*, *msrA*, *ermB*, *mefE*), tetracycline resistance genes (*tetM*), plasmid‐mediated resistance, and *VanA*, *rplD*, and *rplV*	PBP mutations (penicillin resistance), methylation of rRNA (macrolide resistance), *mefE* and *msrA* (macrolide resistance), *tetM* (tetracycline), *VanA* gene (vancomycin resistance), and mutations in *rplD* and *rplV* (ribosomal resistance)	[133, 134, 135, 136, 137, 138]
*C. difficile*	Fluoroquinolones, clindamycin, metronidazole, vancomycin, rifampin, tetracyclines, β‐lactams, and macrolides	Target site mutations (*gyrA*/*gyrB*), ribosomal mutations, efflux pumps, altered enzyme pathways (TdcA and TdcB), rpoB mutations, multidrug efflux (cdeA), *Van* genes, *TetM* and *TetW* genes, and altered PBPs	Mutations in *gyrA*/*gyrB* (fluoroquinolones resistance), ribosomal methylation (clindamycin resistance), efflux pumps (cdeA resistance), *rpoB* mutations (rifampin resistance), *TetM* and *TetW* genes (tetracyclines), altered PBPs (β‐lactams resistance), *Van* genes (vancomycin resistance), overproduction of *TcdA*/*TcdB* toxins, and plasmid‐mediated resistance	[116, 117, 118, 119, 120, 121, 122, 123]
*M. tuberculosis*	Isoniazid, rifampin, pyrazinamide, ethambutol, fluoroquinolones, aminoglycosides, linezolid, bedaquiline, delamanid, and streptomycin	*KatG*, *rpoB*, *pncA*, *embB*, *gyrA*/*gyrB*, ribosomal target, *atpE*, *fbiA*/*fbiC*, and *rrs* mutations	Mutations in *KatG* (isoniazid resistance), *rpoB* gene (rifampin resistance), *pncA* (pyrazinamide resistance), *embB* (ethambutol resistance), *gyrA* and *gyrB* (fluoroquinolones resistance), *rrs* and eis (aminoglycosides resistance), ribosomal binding site (linezolid resistance), *atpE* (bedaquiline), *fbiA*/*fbiC* (delamanid resistance), *rrs* (streptomycin resistance)	[71, 72, 73, 74, 75, 76, 77, 78, 79, 80]

*Note: Helicobacter pylori* (*H. pylori*), *Neisseria gonorrhoeae* (*N. gonorrhoeae*), *Salmonella enterica* (*S. enterica*), *Haemophilus influenzae* (*H. influenzae*), *Enterobacter cloacae* (*E. cloacae*), *Streptococcus pneumoniae* (*S. pneumoniae*), *Clostridioides difficile* (*C. difficile*).

### 3.1 Physiological Adaptations

Physiological adaptations are critical survival strategies that enable diverse microbial pathogens to endure antimicrobial stress and persist in hostile environments. These adaptations involve a spectrum of coordinated responses, including the development of physical barriers, activation of global stress response systems, modulation of metabolic activity, phenotypic heterogeneity, and the formation of biofilms [18, 44, 145]. These mechanisms enable bacteria to survive adverse conditions and modulate their physiological state, enhancing their adaptability, evading immune responses, and overcoming antimicrobial treatments.

#### 3.1.1 Barrier Formation and Stress Response Systems

Bacterial pathogens may withstand antibiotic pressure and environmental stress through fundamental physiological adaptations, such as the creation of barriers and stress response systems. The outer membrane contains a leaflet rich in LPS, selective porins, and efflux channels. These components create a permeability barrier that Gram‐negative bacteria employ to evade antibiotics such as β‐lactams, fluoroquinolones, and aminoglycosides [37, 38, 146]. The outer membrane's porin‐mediated selectivity limits the entry of large or hydrophilic molecules, resulting in innate resistance to many antimicrobial agents. Some Gram‐positive bacteria, such as MRSA, may resist antibiotics because their peptidoglycan layers are thicker, their teichoic acid composition differs, or their surface proteins are altered, making them less susceptible [147, 148].

Mycobacteria, such as *M. tuberculosis*, have a waxy cell wall rich in mycolic acids. This cell wall makes hydrophilic drugs more difficult to penetrate, slowing the development of mycobacteria and rendering them naturally drug resistant [147, 148]. These structural changes across bacterial taxa underscore the importance of bacterial cell membrane composition in intrinsic antibiotic resistance. Antibiotic exposure, oxidative stress, nutritional deficiency, thermal shock, and physical barriers cause bacteria to activate various stress response mechanisms. The stringent response is a key adaptive mechanism that activates in response to nutrient deprivation or antibiotic stress, producing translational stress. Alarmone molecules (p)ppGpp are created during this process. These compounds alter global transcription, reducing energy‐intensive biosynthetic activities (such as replication and translation) while enhancing stress–survival pathways [149, 150, 151]. This response promotes survival in resource‐limited conditions and alters the structure of biofilms, enabling them to endure when exposed to antibiotic stress [18, 152, 153]. The heat shock response is another key mechanism shared by all bacteria, regardless of whether they are Gram‐positive or Gram‐negative. When exposed to antibiotics or temperature changes that induce protein misfolding, bacteria upregulate heat shock proteins (HSPs) such as DnaK, GroEL, and ClpB to aid in the refolding of denatured proteins and maintain protein homeostasis. These chaperones regulate virulence factors and multidrug resistance pathways, enabling bacteria to withstand host immune responses or antibiotic‐induced stress [154, 155]. The HSPs regulate transcription factors that control genes involved in the global stress response in pathogens such as *M. tuberculosis* and *Listeria monocytogenes* (*L. monocytogenes*).

Bacteria possess an innate defensive mechanism known as the oxidative stress response, which they utilize to combat reactive oxygen species (ROS) generated by the host immune system or cellular metabolic activities. Bacteria create peroxidases, catalase, and superoxide dismutase to neutralize ROS and prevent DNA, protein, and lipid damage [149, 156]. Because oxidative stress may stimulate the creation of multidrug efflux pumps, which remove drugs from the cell before their concentrations become inhibitory, this response affects antibiotic resistance. *P. aeruginosa* develops resistance to several drug classes when the *MexAB–OprM* pump is overexpressed [157, 158]. Efflux‐mediated defenses are highly conserved, as evidenced by homologous systems such as *NorA* in *S. aureus* and *LmrP* in *Lactococcus lactis*, which function in Gram‐positive species [159, 160]. Plasmids, integrons, and transposons are mobile genetic elements that facilitate the transmission of resistance genes across organisms. This strengthens physiological defense. Bacteria can adapt and survive under selection pressure because these genetic components influence antibiotic resistance in real‐time via stress response pathways [37, 52]. These mechanisms cause the coselection of resistance and virulence features in *E. faecalis*, *K. pneumoniae*, and *M. tuberculosis*, making infection management and treatment more challenging.

Bacteria use stress response mechanisms and barrier‐building as complementary defenses against antimicrobial threats. They all act as physical barriers to antibiotics, although the structural elements that do so vary among microbial species. Gram‐negative bacteria have outer membranes, mycobacteria have layers of mycolic acid, and Gram‐positive bacteria have thicker peptidoglycan. Bacteria can maintain equilibrium, resist immune system clearance, and withstand therapy because these barriers work with dynamic stress responses, such as thermal shock, oxidative stress, and stringent pathways. A comprehensive understanding of these mechanisms across all bacterial species is required to create next‐generation therapies that target these defenses and restore antibiotic efficacy.

#### 3.1.2 Efflux Pump System

Efflux pumps are membrane protein complexes that actively remove toxic substances from bacterial cells, including antibiotics, and therefore play a crucial role in antibiotic resistance across many microbial taxa. These systems, notably common in Gram‐negative bacteria, work with the outer membrane to reduce internal antimicrobial levels and promote MDR [148, 161, 162]. However, efflux pumps are not exclusive to Gram‐negative organisms. Furthermore, Gram‐positive bacteria, mycobacteria, and certain fungi also play a critical role in maintaining intracellular homeostasis and reducing sensitivity to antibiotics, biocides, and host defense chemicals [38, 163]. The principal families of efflux systems are the resistance‐nodulation‐division (RND) family, major facilitator superfamily (MFS), small multidrug resistance (SMR) family, ATP‐binding cassette (ABC) transporters, and multidrug and toxic compound extrusion (MATE) family, which are classified based on structural characteristics and energy sources [148, 164]. RND‐type efflux pumps are vital for Gram‐negative bacteria. These tripartite complexes utilize the proton motive force to extrude antibiotics, including β‐lactams, fluoroquinolones, tetracyclines, and chloramphenicol [163]. Two noteworthy examples are the AdeABC system in *A. baumannii* and the MexAB–OprM system in *P. aeruginosa*, which are essential for resistance in clinical isolates [38, 158]. MFS and ABC transporters are crucial to Gram‐positive bacteria. *EmeA* in *E. faecalis* and *NorA* in *S. aureus* confer resistance to fluoroquinolones and other drugs by expelling them from the cytoplasm [165]. Efflux pumps, such as Rv1258c, are often activated in response to antibiotic pressure, assisting mycobacteria, particularly *M. tuberculosis*, in developing resistance to isoniazid, rifampicin, and ethambutol [156].

In addition to antibiotic efflux, these systems govern various physiological functions. They aid in the release of virulence factors, quorum sensing (QS), biofilm formation, and responses to environmental stress. The MexEF–OprN system in *P. aeruginosa* regulates quorum‐sensing mechanisms and biofilm formation, thereby increasing survival at subinhibitory antibiotic doses and facilitating the persistence of chronic infections [165]. Efflux pumps help Gram‐positive and mycobacterial species colonize, persist, and evade the immune system. Overexpression of efflux pumps, notably *MexAB–OprM*, hinders infection control strategies in clinical settings by increasing resistance to disinfectants, antiseptics, and antibiotics [166, 167]. Due to their role in multidrug resistance, efflux pump inhibitors (EPIs) are being suggested as adjuncts to enhance the effectiveness of antibiotics. Although several EPIs, including phenylalanine–arginine β‐naphthylamide, have shown promise in vitro, their clinical relevance is limited due to selectivity, toxicity, and pharmacokinetic instability [168, 169]. Nonetheless, advances in high‐throughput screening and structure‐based drug design are driving the search for next‐generation EPIs that simultaneously target multiple efflux systems. Resistance develops and spreads owing to efflux mechanisms. Resistance genes that code for efflux pumps, which are often found on integrons, transposons, or plasmids, facilitate HGT across microbial species [163]. Rapid adaptation and coselection of efflux‐mediated resistance with other resistance traits, such as β‐lactamase production or target site modifications, are possible due to genetic mobility. The global AMR pandemic is exacerbated by environmental reservoirs, including wastewater, soil microbiomes, and agricultural runoff, which serve as centers for the enrichment and dissemination of efflux‐related resistance genes [170].

In conclusion, efflux pumps are multipurpose, conserved processes found in all bacterial phyla and are essential for both acquired and intrinsic antibiotic resistance. They are critical for bacterial adaptability and persistence due to their ability to resist a wide range of drugs and their integration into networks that influence virulence, stress response, and genetic mobility. Understanding efflux pump mechanisms, regulation, and evolutionary dynamics in different bacterial groups is critical for developing new therapeutic strategies, such as the systematic design of broad‐spectrum EPIs and combination therapies to treat MDR infections.

#### 3.1.3 Biofilm Formation

Biofilm development is a vital physiological adaptation that significantly enhances antibiotic resistance in several bacterial species. A biofilm is an organized assemblage of bacterial cells encased in a self‐generated matrix of extracellular polymeric substances (EPS) that adhere to surfaces and to each other [171, 172]. The EPS matrix, composed of proteins, lipids, polysaccharides, and extracellular DNA, acts as a barrier that inhibits antibiotic penetration, shields bacteria from the immune system, and facilitates their survival in adverse environments. Consequently, biofilm‐associated infections are notoriously difficult to eradicate and are often linked to recurring and chronic disease conditions. The heightened antibiotic resistance observed in biofilm bacteria may be attributed to several processes. The deeper levels of the biofilm exhibit diminished antibiotic effectiveness due to the thick EPS matrix, which impedes the diffusion of antimicrobial drugs [173, 174]. Second, oxygen gradients and nutritional scarcity induce bacterial cells to enter low‐energy, slow‐growing, or inactive states, rendering antibiotics that target active processes less effective; therefore, metabolic variability within biofilms contributes to their survival. A subpopulation of phenotypically tolerant cells, termed persister cells, exhibits significant antibiotic tolerance without demonstrating genetic resistance. Chronic infections and therapeutic failure may arise from these cells’ ability to endure therapy and proliferate after antibiotic pressure is alleviated [175, 176, 177].

Biofilms enhance defensive mechanisms by stimulating efflux pump systems. *P. aeruginosa*, in biofilm states, often overexpresses efflux pumps such as *MexAB–OprM*, which aggressively expel antibiotics like β‐lactams and fluoroquinolones, reducing intracellular drug concentrations [159, 160, 178]. By interacting with the biofilm matrix, these mechanisms enhance bacterial survival under antibiotic stress. QS is a signaling mechanism based on cell density that tightly governs biofilm growth by modulating the expression of genes associated with virulence, biofilm maturation, and EPS synthesis [18, 179]. For example, QS pathways such as *Las*, *Rhl*, and *PQS* in *P. aeruginosa* regulate biofilm formation and promote the secretion of virulence proteins that exacerbate infections and facilitate immune evasion [180]. The high cell density of biofilms facilitates collective decision‐making, rendering the bacterial population more resilient to host defenses and external threats such as antibiotics. *S. aureus* and *E. faecalis* exemplify Gram‐positive bacteria capable of forming robust biofilms, particularly on host tissues and medical devices. The *icaADBC* gene cluster in *S. aureus* regulates the production of polysaccharide intercellular adhesin, which is essential for EPS synthesis and biofilm stability. *M. tuberculosis* may form biofilm‐like structures during lung infections, contributing to its chronicity and resistance to anti‐TB drugs [181, 182].

Persister cells—metabolically dormant cells capable of enduring elevated antibiotic concentrations and later reinitiating infection—constitute a significant therapeutic challenge associated with biofilms. In contrast to genetically resistant cells, persisters emerge randomly and return to a vulnerable condition whenever the antibiotic is withdrawn. Antibiotic research and medication development are increasingly focused on them because of their involvement in treatment failure and relapse [176, 177]. Moreover, biofilms act as focal points for HGT. The proximity of various microbial cells and the availability of extracellular DNA create optimal conditions for exchanging resistance genes through transformation, conjugation, or transduction [123, 183]. Antibiotic resistance may proliferate more rapidly in mixed‐species biofilms due to the exchange of mobile genetic elements, such as integrons and plasmids, between commensal and pathogenic bacteria [18, 184]. Biofilms on medical equipment, including ventilators, prosthetics, and catheters, serve as enduring reservoirs for MDR microbes, posing significant concerns in healthcare environments [16].

In summary, biofilm formation is a sophisticated resistance mechanism that enhances bacteria's survival, persistence, and adaptability. It enables bacterial colonies to resist drugs and evade immunological responses by integrating QS, metabolic dormancy, efflux pump overexpression, and physical protection through the production of EPS. Due to these characteristics, biofilms provide a considerable obstacle to managing chronic and device‐related diseases. There is an immediate need for innovative treatment strategies that address persister cells, disrupt QS, and compromise biofilm integrity. Despite the potential of EPIs and quorum‐sensing inhibitors, their clinical advancement is hindered by pharmacokinetic challenges, potential toxicity, and insufficient selectivity [168, 169]. Current research on biofilm‐specific processes offers promising prospects for enhancing the efficacy of conventional antibiotics and reducing the global incidence of biofilm‐related antibiotic resistance.

#### 3.1.4 Coccoid Formation

The formation of coccoid structures is a crucial morphological and physiological adaptation in bacteria, enabling them to survive in challenging environments such as antibiotic pressure, oxidative stress, food scarcity, and host immune responses. Rod‐shaped or spiral bacteria generally convert into spherical, inactive coccoid forms that are more resistant to antimicrobial drugs, exhibit lower metabolic activity, and may evade the immune system [147, 176, 185]. This adaptation has been observed in numerous taxa. It is a crucial survival strategy in chronic and recurring infections, although it has been most extensively studied in specific Gram‐negative bacteria, such as *H. pylori* and *C. jejuni*.

Environmental stressors often encountered in the gastrointestinal tract, including antibiotic exposure, oxidative damage, and nutritional deprivation, cause coccoid transformation in *C. jejuni*. The coccoid form is metabolically inert and resistant to antibiotics that target active cellular processes, such as β‐lactams and fluoroquinolones [186, 187]. This form enables bacteria to evade the immune system, allowing them to survive within the host and reactivate when conditions improve. Chronic or recurrent *C. jejuni* infections are primarily caused by a switch from a coccoid to a spiral shape [188]. *H. pylori* uses coccoid conversion to persist in the acidic environment of the human stomach, especially during antibiotic treatment. *H. pylori*’s resistance to antibiotics, such as macrolides and fluoroquinolones, is enhanced by changes in the 23S rRNA and *gyrA* genes, which reduce metabolic activity [189, 190, 191]. Furthermore, coccoid *H. pylori* cells may resist eradication during standard treatment procedures due to their increased resilience to acidic stress and host proteolytic enzymes [192, 193]. When antibiotic pressure is removed, coccoid cells may revert to their spiral, replicative form, potentially leading to reinfection and the recurrence of ulcers or chronic gastritis [194, 195].

Coccoid transformation, although most commonly found in Gram‐negative pathogens, is also observed in other bacterial species with similar morphological plasticity. When exposed to prolonged environmental or antibiotic stress, some Gram‐positive cocci and mycobacteria may become nonculturable or latent, resembling coccoids. *S. aureus*, *S. pneumoniae*, and *E. faecalis* are Gram‐positive organisms that take on small, metabolically inactive forms in response to pharmacological stress or chronic infections [196, 197, 198]. *Mycobacterial* species, such as *M. tuberculosis* and *M. smegmatis*, can adopt nonreplicating, persistent states in response to hypoxia, food restriction, or pharmaceutical therapy, resulting in decreased or coccoid‐like shapes [199, 200, 201]. The long lifespans of these organisms are attributed to their stiff membranes, oxidative stress resistance genes, and persistently low metabolic rates [202, 203, 204, 205]. Nonetheless, the difficulties in detecting coccoid and dormant cells using conventional diagnostic techniques based on culturability or metabolic activity may be overlooked during treatment, potentially leading to underdiagnosed persistence and poor therapeutic outcomes [44, 206, 207, 208].

Coccoid cells often cohabit with persister cells in biofilms and host habitats, with the latter being a distinct phenotypic variety characterized by transient antibiotic resistance [209, 210]. Coccoid forms, such as persisters, are resistant to elimination due to their physiological state; however, they lack specific resistance genes [211, 212]. Dormant *S. aureus* cells in biofilms may evade immune responses and antimicrobial therapies, leading to recurring infections in the skin, bones, and catheters [196, 213]. Bacterial populations in chronic conditions may acquire antibiotic resistance, complicating the treatment of infection [214]. The coccoid shape promotes environmental adaptation. *C. jejuni*’s coccoid metamorphosis increases survival in nutrient‐deficient food and water sources while improving intestinal oxidative stress resistance [186, 215]. Coccoid *H. pylori* has been identified in wastewater and on hospital surfaces, suggesting its possible involvement in indirect transmission [191]. Dormant *M. tuberculosis* bacilli within pulmonary granulomas may remain quiescent for decades, reactivating only when host immunity is weakened. This reveals that coccoid forms have significant public health implications as environmental reservoirs that facilitate reinfection and the spread of antibiotic resistance, in addition to serving as host survival strategies.

Ultimately, the coccoid transformation is a reversible survival strategy essential for antibiotic resistance, bacterial persistence, and prolonged infection. The morphological change enables bacteria to evade detection, elude immune clearance, and tolerate antibiotic treatment, regardless of genetic resistance mechanisms. When antibiotic pressure is removed, latent forms may reawaken and become virulent, leading to treatment failure and recurrent infections. This emphasizes the need to include morphologically changed forms—such as coccoids and other latent phenotypes—in antimicrobial screening, treatment, and diagnostic protocols. Due to their clinical significance and diagnostic ambiguity, coccoid forms must be considered when developing novel diagnostic tools, antimicrobial drugs, and treatment methods to eradicate persistent infections and prevent antibiotic resistance.

#### 3.1.5 Altered Growth Rates

Bacteria may alter their growth rate in response to stress. This significant physiological alteration renders several bacterial species resistant to antibiotics. When subjected to drugs, oxidative stress, or nutritional deprivation, bacteria can transition from active replication to a state of sluggish growth or dormancy. This enables them to circumvent the impact of antibiotics that primarily affect rapidly proliferating cells [3, 39, 216]. This capacity to alter their growth and morphology enables bacteria to thrive in clinical and environmental contexts. As bacterial growth diminishes, their metabolic activity also decreases concurrently. This allows them to conserve energy and manage stress for extended periods. *A. baumannii*, a prominent Gram‐negative bacterium responsible for hospital infections, diminishes its metabolic activity in response to significant antibiotic pressure or nutritional deprivation, leading to an increase in its resistance to therapy [217, 218]. This metabolic alteration is often associated with the heightened synthesis of β‐lactamase enzymes and efflux pumps, which actively extrude antibiotics from the bacterial cell, thereby enhancing resistance to numerous antibiotics [176, 218].

The production of small colony variants in Gram‐positive bacteria, such as *S. aureus*, is associated with reduced growth rates [213, 219]. This enables the bacteria to persist inside the host and evade antibiotics during infection. Mycobacteria, particularly *M. tuberculosis*, employ delayed reproduction as a natural defense mechanism [220]. The pathogen may assume a nonreplicative persistent condition inside granulomas, making it more drug resistant. This complicates the treatment of TB [39]. Bacteria may also synchronize alternative mechanisms of resistance as their growth conditions change. The stress response systems, biofilm development, and efflux pump regulation impede metabolic processes. In *P. aeruginosa*, food deprivation enhances the activity of the MexAB–OprM efflux pathway [178]. This improves the bacteria's resistance to β‐lactams and fluoroquinolones, allowing them to survive amid fluctuations in antibiotic concentrations [18, 160, 167].

Furthermore, populations that decelerate their metabolism or cease development often include persister cells, phenotypic variants capable of surviving antibiotic treatment without genetic resistance [221, 222]. These cells are frequently located in biofilms, which enter a dormant state due to a lack of oxygen or nutrients. These latent cells may reactivate after the antibiotic threat subsides, potentially leading to recurrent infections [177, 223, 224]. The transition from an active to a dormant phenotype complicates the management and eradication of diseases. Bacteria may alter their growth rate, a significant adaptation that enhances their drug resistance and facilitates their evolution [223]. This approach enables bacteria to persist inside the host, withstand eradication during therapy, and revive when circumstances become favorable. The alteration in growth rate, along with modifications in metabolism, efflux activity, and persistence, renders standard antibiotics ineffective [225]. To address this issue, we need novel methods to target actively dividing bacteria and slow‐growing or dormant microorganisms. Specific techniques may include pharmaceuticals that inhibit metabolic dormancy, enhance the immune system's ability to detect dormant cells, or synergize with current antibiotics to eliminate persisters and reduce the likelihood of recurrence.

### 3.2 Genetic Foundation

Genetic alterations are crucial for bacterial survival and the proliferation of AMR. Point mutations, HGT, and gene amplification are critical genetic mechanisms that enable bacteria to rapidly adapt to antibiotic pressure. These activities facilitate the emergence and dissemination of resistance genes such as β‐lactamases, efflux pump regulators, and changes in antibiotic targets. Recent advancements in whole‐genome sequencing (WGS) and NGS have enhanced our comprehension of genes and pathways associated with resistance. These instruments have significantly improved our understanding of bacterial evolution and the acquisition of resistance characteristics in clinical and environmental contexts [226, 227]. This illustrates the genetic complexity and adaptability of AMR.

#### 3.2.1 Origins and Evolution of Genetic Mutations

Mutations in genes are the primary source of AMR in bacteria, including Gram‐positive and Gram‐negative pathogens [39]. Some mutations arise naturally during DNA replication, whereas others are caused by environmental stresses such as oxidative damage, antibiotics, or mutagenic chemicals. In the face of antibiotic‐induced selection, mutations that provide resistance allow clones to proliferate and disseminate more easily. Antibiotic efficacy is reduced due to chromosomal alterations in regulatory components, efflux systems, and target sites. It is well known that fluoroquinolone‐resistant bacteria often have point mutations in the genes encoding DNA gyrase (*gyrA*) and topoisomerase IV (*parC*). Additionally, substituting Ser83 and Asp87 in *GyrA* and Ser80 and Glu84 in *ParC* reduces the quinolone binding affinities of *S. enterica*, *P. aeruginosa*, and *E. coli* [14, 228]. As a result of the upregulation of efflux systems, such as AcrAB–TolC, and an increase in resistance across several drug classes, mutations in global regulators, such as marA or soxS, sometimes occur alongside these target alterations [159, 226].

In β‐lactam resistance, an increase in enzyme production can be caused by mutations in the coding sequences or promoter regions of β‐lactamase genes, including *bla_TEM*, *bla_CTX‐M*, or *bla_OXA* [229]. In A. *baumannii*, *bla_OXA* variants become hyperexpressed when promoter alterations or insertion sequence activation, especially with IS elements like ISAba1, occur [230, 231]. *K. pneumoniae* is partly resistant to carbapenems because mutations in *OmpK35* and *OmpK36* reduce permeability and promote porin loss, which is especially significant when combined with carbapenemase production. Inserting into loop 3 of *OmpK36* can effectively exclude β‐lactams [226, 232]. The *mecA* gene mediates methicillin resistance in Gram‐positive bacteria such as *S. aureus*. PBP2a is a PBP with a decreased affinity for β‐lactam antibiotics [233, 234]. This gene is part of the mobile SCCmec cassette, which can be transferred horizontally and often includes additional resistance genes [234]. Transposable elements, such as IS256, also facilitate gene movement [235]. In order to make *E. faecium* resistant to vancomycin, the vanA and vanB operons alter cell wall precursors and reduce glycopeptide binding [236].

*N. gonorrhoeae* is a model of cephalosporin resistance due to mosaic mutations in *penA*, particularly changes in the PBP2 domain that decrease drug binding, such as the PenA‐60 variant [237, 238]. Mutations in *mtrR*, the mtrCDE efflux pump operon's repressor, result in derepressed efflux and enhanced resistance [40, 226]. Resistance complexity increases when MDR organisms, such as *S. enterica*, *P. aeruginosa*, and *K. pneumoniae*, accumulate synergistic mutations in the *gyrA*, *parC*, *acrB*, and regulatory loci [239, 240]. Treatment is further complicated since acquired resistance determinants often exist alongside these mutant combinations. Finally, environmental or spontaneous changes to structural proteins, efflux regulators, or drug targets are crucial factors in the evolution of resistance. Recent technological advances have enabled the improved prediction of MDR pathogen evolutionary trajectories and the identification of resistance hotspots. These include CRISPR–Cas9, high‐throughput sequencing, and mutation monitoring platforms [226].

#### 3.2.2 Transmission Dynamics Through HGT

Bacterial taxa, including both Gram‐positive and Gram‐negative species, have developed antibiotic resistance due to HGT [241]. HGT enables bacteria to acquire resistance genes from different strains, species, or even distant genera, thereby accelerating evolution and increasing AMR [242]. The three primary mechanisms of HGT—transduction, conjugation, and transformation—substantially impact the dissemination of resistance [243]. In both environmental and clinical contexts, conjugation plays a significant role, facilitating the transfer of plasmid‐borne multidrug resistance traits [4, 226]. Colistin and carbapenem resistance genes such as *bla_KPC*, *bla_NDM‐1*, and *mcr‐1* can be transmitted using conjugative plasmids from families including IncX3, IncHI1, and IncFII [244, 245]. Polyresistant plasmids, which comprise arrays of genes that confer resistance to aminoglycosides, fluoroquinolones, and β‐lactams, contribute to the global spread of MDR strains. Many Gram‐positive bacteria, including *S. aureus* and *E. faecalis*, rely on conjugative transposons, such as Tn916, to transmit genes that confer resistance to tetracyclines and macrolides.

Integrated sequences, transposons, and plasmids are not the only mobile genetic elements that can mobilize resistance genes. One example is the *bla_KPC* gene, which can be inserted into different plasmids or chromosomal loci due to the Tn4401 transposon [246, 247]. Gene cassettes confer resistance to β‐lactams, sulfonamides, and aminoglycosides, which are expressed and captured by Class 1 integrons, as seen in *Salmonella*, *K. pneumoniae*, and *E. coli* [248]. ICEs mediate the chromosomal integration of resistance genes while maintaining conjugative mobility; examples of such elements include the SXT/R391 family in Vibrio cholerae and the Tn916‐type ICEs in Gram‐positive bacteria [249, 250]. Notably, even when significant selection pressure is not present, environmental stress can often increase the frequency of plasmid transfers by amplifying HGT, especially at concentrations of sub‐inhibitory antibiotics. In addition, research has shown that strain‐specific activation of the efflux pump enhances plasmid uptake and HGT in both planktonic and biofilm‐associated bacteria [226, 251]. Biofilms provide favorable conditions for gene transfer by increasing cell–cell interactions, protecting against antibiotics, and stabilizing the transferred elements [252]. Antibiotic stewardship, infection prevention, and genetic monitoring strategies to contain the AMR epidemic must be guided by a thorough understanding of HGT dynamics among bacterial species, as shown by these mechanisms.

#### 3.2.3 Gene Amplification and Duplication

Bacteria employ powerful evolutionary mechanisms, such as gene duplication and amplification, to increase their antibiotic resistance [41, 164]. Antibiotic stress causes these processes to increase the copy number of specific resistance genes, enhancing the production of efflux pumps, antibiotic‐modifying enzymes, or altered target proteins. For bacteria, amplification is a rapid and reversible adaptation mechanism that enables them to adjust their resistance levels to various environments. The regulation of the *acrAB–tolC* efflux operon amplification in *E. coli* by transcriptional activators such as *Rob*, *SoxS*, and *MarA* is an example that has been extensively studied [253, 254]. These regulators form an integrated resistance network, which upregulates efflux activity and influences the expression of porin genes and stress response genes [226]. Intracellular concentrations of several antibiotics are significantly decreased when efflux components are overproduced through gene amplification [160, 255].

Bypassing the requirement for plasmid acquisition, a new strategy that utilizes YRIN/YRIK‐type duplications enhances β‐lactam resistance by increasing the dosage of β‐lactamase‐encoding genes [256, 257]. Duplication of the *bla_KPC* and *bla_NDM* genes results in significant carbapenem resistance; this genetic innovation has been identified in both *E. coli* and *K. pneumoniae* [47, 258]. The same type of duplication events has been observed in Gram‐positive bacteria, including mecA amplification in MRSA, which leads to increased β‐lactam resistance [234].

The RND‐type efflux systems are commonly used in A*. baumannii* for resistance amplification, particularly in the context of nosocomial infections [259, 260]. It is essential to follow strict dosing guidelines, as these amplifications may occur after exposure to sub‐lethal amounts of antibiotics. Bacteria frequently lose or downregulate excess gene copies as antibiotic pressure subsides, complicating diagnostic identification and resistance prediction. Studies have demonstrated that gene amplifications may be genetically unstable [261, 262, 263]. Developments in NGS and WGS have revealed genomic hotspots linked to amplification events, which could serve as diagnostic or therapeutic targets [227, 264]. To comprehend bacterial adaptation, it is essential to identify the regulatory networks and mobile components that mediate these processes. In summary, resistance mechanisms in bacteria are dynamic and dose dependent, involving the amplification and duplication of genes. They contribute to the complexity of multidrug resistance while offering a reversible and energy‐efficient approach to addressing antimicrobial threats. To combat these pathways, it will be necessary to develop drugs that can bypass or inhibit amplification‐driven resistance. This will require the integration of techniques, including genomics, pharmacology, and molecular microbiology.

### 3.3 Biochemical Mechanisms of Resistance

Antibiotic resistance is due to biochemical mechanisms that involve various metabolic and molecular modifications that bacteria use to survive when faced with antibiotic pressure. Some examples include altering the structure of pharmacological targets, reconfiguring key metabolic pathways, or modifying antibiotics enzymatically. When these tactics are combined, they significantly contribute to the spread and persistence of antibiotic resistance [44, 227].

#### 3.3.1 Enzymatic Inactivation

As one of their most effective biochemical survival mechanisms, bacteria use various enzymatic techniques to counteract antibiotics. Antibiotics can be rendered ineffective by enzymatic inactivation, chemical alteration, or the breakdown of antibiotic molecules [41, 265]. The mechanisms that have been investigated the most intensively are β‐lactamases. These enzymes disrupt the β‐lactam ring of many antibiotics, rendering them unable to bind to PBPs [47, 266]. PBPs play a crucial role in the production of bacterial cell walls. Based on whether they utilize a serine residue or divalent metal ions (Zn^2^⁺) for catalysis, β‐lactamases are structurally classified as either metallo‐β‐lactamases or serine β‐lactamases [267, 268]. It is common for clinical isolates of ESBLs, such as the bla_CTX‐M_ gene family, to be present in *E. coli*, *K. pneumoniae*, and *S. enterica*. This gene family can hydrolyze third‐generation cephalosporins [45, 269]. There has been an increase in resistance in clinical settings due to the emergence of carbapenemases in Gram‐positive and Gram‐negative bacteria, including *Enterococcus* species [270].

Enzymatic inactivation not only affects β‐lactams but also other types of antibiotics. The process of protein synthesis can be hindered by aminoglycoside‐modifying enzymes, such as acetyltransferases, phosphotransferases, and nucleotidyltransferases [271, 272]. The phosphorylation of hydroxyl groups on aminoglycoside rings is catalyzed by enzymes like APH(3′)‐IIIa and APH(6′)‐IIa. This tactic is employed by both Gram‐positive and Gram‐negative bacteria, including *S. aureus* and *E. faecalis* [273, 274]. The aac(3)‐II gene is commonly found in *K. pneumoniae* that is resistant to gentamicin and tobramycin [275]. The chloramphenicol acetyltransferase is another key example; it facilitates resistance by acetylating chloramphenicol, thereby preventing it from binding to the 50S ribosomal subunit [276, 277, 278]. According to recent studies [279, 280, 281], plasmids, transposons, and integrons are common carriers of these enzymatic changes, enabling their horizontal transmission and rapid dispersion among bacterial populations.

Resistance escalation is aided, crucially, by enzyme variability and point mutations. One example is how NDM‐type metallo‐β‐lactamases can hydrolyze newer β‐lactams by altering their active sites [282]. Enzyme inhibitors such as avibactam, relebactam, and vaborbactam have been developed more rapidly through structural research and in silico modeling. These drugs are used in combination with β‐lactam antibiotics [283]. In addition, the SOS response and enzymatic inactivation often function together, accelerating the emergence of mutations that confer antibiotic resistance and enhancing mutagenesis [227, 284]. Metabolic rewiring, which involves changes in carbon utilization and redox balance, indirectly reduces drug efficacy and supports bacterial adaptation [285, 286]. Enzymes that control redox homeostasis or amino acid biosynthesis may undergo structural changes, which can decrease antibiotic absorption or target engagement [222, 287].

Overall, one of the most common and effective resistance mechanisms in bacteria is enzymatic inactivation, which encompasses processes such as degradation, phosphorylation, and acetylation, as well as their incorporation into larger metabolic and stress–response networks. When designing new inhibitors and antimicrobials, it is crucial to have a thorough understanding of the structural biology, genetic mobility, and regulatory environments of these enzymes.

#### 3.3.2 Metabolic Pathway Adaptations and Bypass

Bacterial pathogens, regardless of their Gram categorization, possess metabolic mechanisms that are remarkably flexible, enabling them to adapt structurally and functionally to withstand antibiotic treatment. Modifying central metabolic pathways in response to antibiotic exposure is a commonly recognized mechanism that reduces the efficacy of antibiotic treatments [265, 285]. To reroute biosynthetic pathways or alter cofactor production, these adaptations can compromise antibiotic targets or establish new pathways for synthesizing essential metabolites. For instance, it has been found that resistance to sulfonamides can be attributed to the acquisition of alternative dihydropteroate synthase (DHPS) enzymes encoded on plasmids by various bacterial species [288, 289]. Folate biosynthesis, a pathway essential for nucleotide synthesis, DNA replication, and energy metabolism, is maintained by this enzyme, allowing it to evade sulfonamide suppression [157].

Similarly, mutations or overproduction of the folate pathway enzyme dihydrofolate reductase (DHFR) are significant causes of trimethoprim resistance. These modifications ensure cell survival and proliferation by reducing the binding affinity of trimethoprim, thereby allowing continued folic acid synthesis [279, 290]. Not only can Gram‐negative bacteria undergo metabolic remodeling, but so can Gram‐positive pathogens, including *Staphylococcus* and *Streptococcus* spp. More recent research has demonstrated that changes in bacterial metabolism play a greater role in the development of antibiotic resistance than previously thought [284]. Modulating redox homeostasis is one example of an adaptation; others include changes to glycolysis and the pentose phosphate system. To adapt to environments exposed to drugs, certain bacteria, such as *K. pneumoniae* and *M. tuberculosis*, modify their sugar degradation and energy metabolism processes [40, 291].

Another metabolic hub that is often altered when antibiotics are used is the production of cell walls. A decrease in β‐lactam binding affinity causes resistance when PBPs are overexpressed or structurally modified, particularly in MRSA and resistant strains of *Enterococcus* [292, 293]. Take PBP2a as an example; it continues to synthesize peptidoglycan even when exposed to high doses of β‐lactam. This mechanism works in tandem with decreased membrane permeability and active drug efflux to produce a phenotype of multifactorial resistance [280]. Mutations in the catalase‐peroxidase gene *katG* alter the oxidative stress response and mycolic acid synthesis, contributing to isoniazid resistance in *M. tuberculosis* [294, 295]. These genetic alterations enable infections to resist oxidative damage and persist in harsh, antibiotic‐contaminated environments [185, 296].

Bacteria may adapt to low oxygen levels and oxidative stress, which makes them more persistent. For example, *E. coli* can adapt to antibiotic stress by switching to anaerobic metabolism when exposed to nitrofurantoin [297, 298]. This process lowers intracellular ROS. Metabolic plasticity is employed by facultative anaerobes, such as Enterobacteriaceae and lactic acid bacteria, to avoid oxidative damage caused by antibiotics [299, 300]. This metabolic flexibility is seen in many bacterial species, not only Gram‐negative ones. Similar modifications are made by Gram‐positive organisms, such as *C. difficile* and *L. monocytogenes*, to their core metabolic pathways to survive in environments rich in drugs [284, 301]. The evolutionary benefit of metabolic flexibility is highlighted by such extensive adaptation.

Ultimately, one of the most critical factors in antibiotic resistance is the metabolic adaptability of bacteria. These alterations, which encompass cell wall production, folate metabolism, anaerobic respiration, and oxidative stress mitigation, allow bacteria, whether Gram‐positive or Gram‐negative, to withstand antimicrobial pressure. We need to understand the metabolic changes that cause resistance to the creation of new antibiotics and diagnostics.

## 4 Interplay of Genetic, Biochemical, and Physiological Systems in Antibiotic Resistance

**FIGURE 3:**
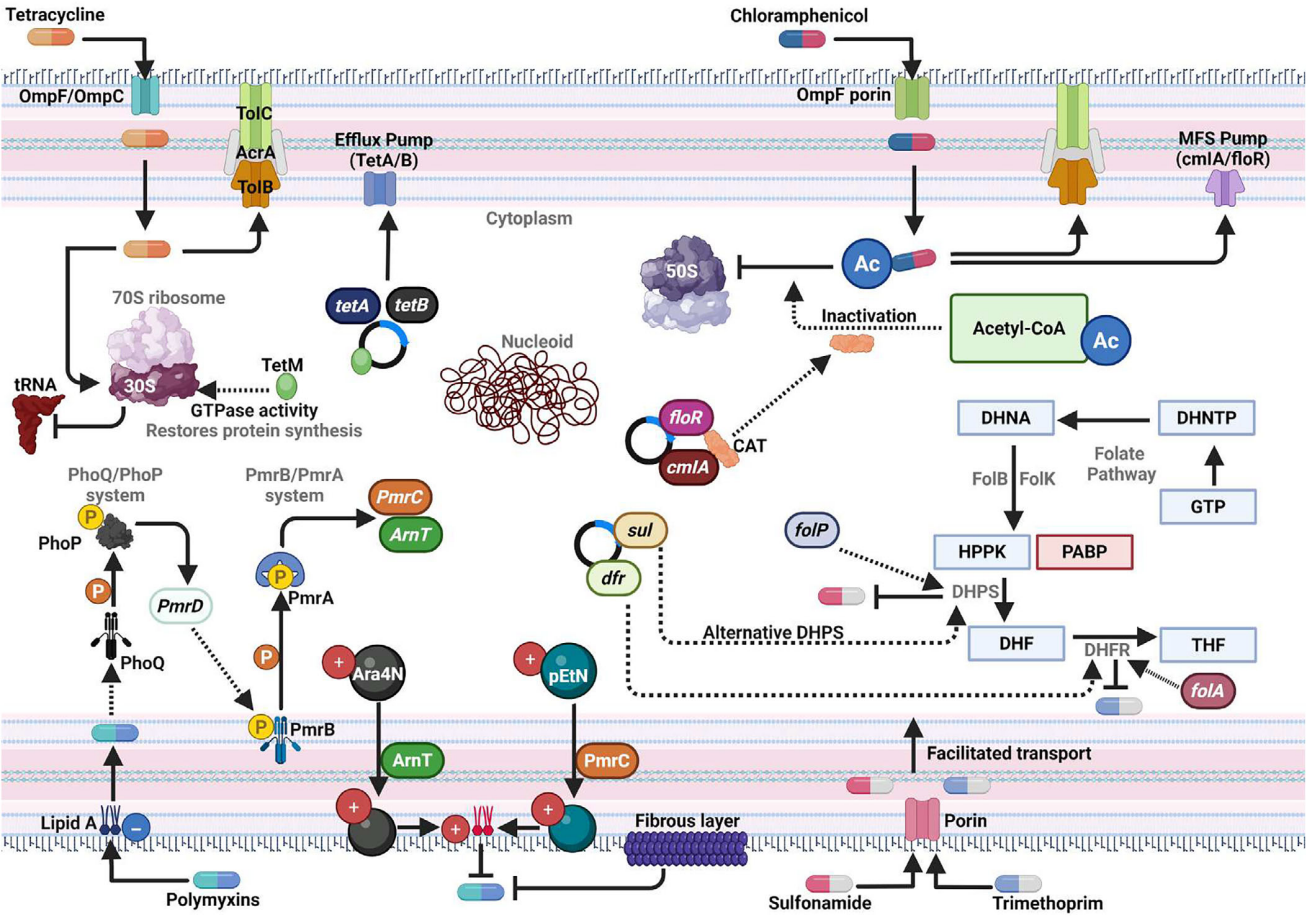
The complex interplay between bacterial genetics, physiology, and biochemistry in the development of antibiotic resistance. The intricate biochemical, physiological, and genetic mechanisms by which Gram‐negative bacteria evade antibiotics are depicted in this picture. The buildup of antibiotics such as tetracycline and chloramphenicol is prevented by active efflux mechanisms, even though they enter the cell through outer membrane porins (e.g., OmpF/OmpC). A system of AcrAB–TolC‐linked TetA/B transporters removes tetracycline, while MFS transporters (cmlA, floR) eliminate chloramphenicol. And to keep protein synthesis going, there's TetM, a GTPase‐active ribosomal protection protein that moves tetracycline out of the 70S ribosome. A key component of resistance is the deactivation of enzymes. The 50S subunit cannot bind chloramphenicol because it is acetylated by chloramphenicol acetyltransferase (CAT). Polymyxin exposure or low magnesium levels are environmental stresses that trigger the activation of genes like *pmrC* and *arnT* through two‐component systems (PhoQ/PhoP and PmrB/PmrA). Some examples of such genes include *ArnT* and *PmrC*, which code for lipid A‐modifying enzymes that decrease polymyxin binding by reducing the negative charge of the membrane. An additional regulator of this system is the *mgrB* gene. Enzymes other than those targeted by sulfonamides and trimethoprim, which block the folate pathway, are involved in resistance. Sulfonamides inhibit DHPS, whereas trimethoprim targets DHFR. Resistance genes (such as *sul1*/*sul2* for DHPS and *dfrA* for DHFR, or *folP* and *folA*) expressed on chromosomes or plasmids keep folate synthesis going. Upstream of HPPK, enzymes such as FolB and FolK contribute to DHP synthesis from PABA. This complex resistance network comprises effluent removal pumps, enzymatic breakdown, membrane remodeling, and target alteration. Two‐component regulatory mechanisms coordinate these reactions. Resistance genes such as *tet*, *cat*, *sul*, *dfr*, *cmlA*, and *floR* can be horizontally transferred by mobile genetic elements, including plasmids and transposons, which accelerates the development of multidrug resistance in Gram‐negative populations. Figure created using BioRender.

The ability of bacteria to survive and adapt in the face of antibiotic pressure is not caused by separate mechanisms, but rather by a complex web of interactions between their genetic code, biochemistry, and physiology (Figure 3). Both Gram‐positive and Gram‐negative bacteria can produce MDR phenotypes, maintain cellular functions, and evade antibiotics due to the complex network that these systems form [41, 227]. HGT and spontaneous mutation are two common genetic events that can introduce new features, initiating the process of resistance. Nevertheless, acquiring resistance genes cannot guarantee bacterial survival without subsequent biochemical and physiological adaptations. To maximize their resistance potential, a bacterium that acquires a gene coding for β‐lactamase or aminoglycoside‐modifying enzymes must accomplish several tasks simultaneously, including upregulating relevant transcriptional pathways, adjusting energy metabolism, and coordinating efflux responses [285, 302]. Enzymatic changes and regulatory networks that respond to antibiotic exposure and environmental stress are components of these adaptive cascades.

When genetic factors that code for ESBLs, carbapenemases, or altered PBPs promote biochemical resistance by destroying or changing antibiotic targets, the interaction between these factors becomes clear. To maximize the retention and utility of resistance mechanisms encoded in DNA, physiological systems adjust membrane permeability or efflux pump activity [303]. Mutations in genes encoding porins can decrease the permeability of the outer membrane, which, in tandem with enzymatic degradation, limits the uptake of antibiotics [232, 304]. Genetic circuits that detect intracellular antibiotic levels and activate efflux in response are often found to govern the overexpression of efflux pumps, such as *MexAB–OprM* in *P. aeruginosa* or *NorA* in *S. aureus* [47, 158].

Metabolic rewiring also mediates the relationship between antibiotic resistance and changes in gene expression. To maintain nucleotide synthesis while drugs are present, biochemical pathways must adapt when genes related to folate metabolism (e.g., *dhfr* and *dhps*) are altered [305]. In addition to affecting bacterial tolerance, these metabolic changes impact cellular redox status and energy allocation, which, in turn, influence physiological features such as biofilm formation and dormancy [291, 301]. Biofilm environments intensify this tri‐system interaction by encouraging HGT, limiting antibiotic penetration, and producing metabolic quiescence (in which antibiotics are less effective) [276]. By bringing these systems together, stress responses show how bacteria's resistance can be quickly adjusted. An example is how antibiotic treatment frequently triggers the SOS response [306]. This response increases the expression of repair enzymes that counteract mutations and alter cell physiology by stiffening the membrane and enhancing efflux activity [284, 307]. Global transcriptional regulators, such as sigma factors or *LexA*, typically control these reactions, which connect genetic stress sensing to molecular adaptability and physiological remodeling.

Integrity is further enhanced by epigenetic control. Essential resistance genes, such as those encoding efflux pumps or target‐modifying enzymes, can affect their expression through DNA methylation or modification of histone‐like proteins [305]. In response to colistin exposure, for example, lipid A structures can change, illustrating one way epigenetic modifications impact downstream biochemical and physiological activities [308, 309]. This proves that resistance is a controlled and ever‐changing process within cells, rather than a fixed attribute passed down through generations via plasmids or mutations. The positive feedback loops that exist between these systems are crucial. Metabolic and membrane alterations that facilitate the function of resistance elements are frequently upregulated upon genetic acquisition [310]. As a result, bacterial populations’ resistance genes are stabilized and passed down through generations when biochemical detoxification or membrane adaptation is successful. For instance, in *M. tuberculosis*, the *katG* mutation hinders isoniazid activation [311]. However, the bacteria can withstand oxidative and antibiotic stress due to physiological adjustments that enhance their antioxidant defenses and cell wall remodeling [294, 296].

To summarize, the maintenance of antibiotic resistance is achieved through a complex and interdependent mechanism that encompasses genetic, biochemical, and physiological systems. Initiation of resistance occurs at the genetic level, antibiotics are modified or degraded by biochemical pathways, and physiological systems preserve cellular function and environmental reactions. Bacteria can adapt their survival tactics to various situations thanks to their ability to integrate into complex systems. These environments include host tissues, hospitals, and even farms. Thus, it is crucial for therapeutic strategies to consider the interplay between these systems, aiming to inhibit not only specific resistance genes but also the metabolic, regulatory, and structural factors that promote their expression.

## 5 Bridging Preclinical and Clinical Antibiotic Development

Developing new antibiotics is a complex process that begins with preclinical research and continues through clinical application. Each step is crucial because it helps ensure the safety, effectiveness, and mechanistic understanding of the treatments [4, 23]. The pharmacodynamics, pharmacokinetics, and resistance potential of antimicrobial drugs are evaluated in preclinical investigations, which include in vitro analyses and animal models. The effectiveness of drugs can be assessed by simulating human infections in rodent models, which include rats and mice, as well as larger animals like rabbits and nonhuman primates. One example is the use of rabbit and mouse skin infection models to study MRSA, whereas β‐lactam drugs are tested against *E. coli* infections in murine models [312, 313]. These models provide a solid scientific foundation for clinical translation by shedding light on the behavior of pathogens and the activity of drugs in living organisms.

Preclinical studies are crucial for assessing new drugs against a variety of resistant microorganisms, including Gram‐positive pathogens such as *S. aureus*, Gram‐negative species like *P. aeruginosa*, and slow‐growing organisms like *M. tuberculosis*, as the rise of AMR is a worldwide concern [4, 314]. For instance, the hollow‐fiber infection model is used to evaluate the effectiveness of treatments for MDR bacteria, such as rifampicin‐resistant *M. tuberculosis* and carbapenem‐resistant *K. pneumoniae* [315, 316]. It is necessary to develop optimal dosage regimens and strategic combination treatments to tackle complex resistance mechanisms. These mechanisms include active efflux, enzymatic antibiotic degradation, and biofilm‐mediated protection. Modern preclinical infection models that mimic the microbial and physiological complexity of resistant diseases allow for comprehensive testing.

Antimicrobial peptides (AMPs) and bacteriophage therapy are two of the most talked‐about potential alternatives to traditional antibiotics. In vivo models of wound infections caused by *S. aureus* and *pneumonia* induced by *A. baumannii* have been tested using these agents [317, 318]. To increase antibiotic uptake, AMPs compromise the integrity of bacterial membranes. Research has shown that AMPs and antibiotics such as β‐lactams and fluoroquinolones can work together to treat resistant *S. pneumoniae* and *P. aeruginosa*, suggesting that these two classes of drugs could be beneficial as adjuvant treatments [319]. Since phages can selectively enter the extracellular matrix and lyse embedded bacterial cells, they have also been effectively used to treat biofilm‐associated diseases. Additionally, nanotechnology‐based drug delivery systems are being developed to overcome physical and biological barriers, thereby enhancing the bioavailability of antibiotics. Evidence suggests that nanoparticles, such as lipid‐based formulations or polymeric carriers, can enhance drug accumulation at infection sites, facilitate translocation across bacterial membranes, and promote sustained release [320, 321]. The therapeutic efficacy of antimicrobial agents is enhanced by these technologies, which also aid in overcoming active efflux mechanisms and permeability barriers. Taken as a whole, these novel approaches represent a game‐changer for preclinical antibiotic research, opening up exciting new possibilities for the precise targeting of treatments against MDR pathogens.

To predict the development of resistance and drug–target interactions, computational modeling has become essential to preclinical research. Machine learning algorithms can mimic natural selection by combining genetic and phenotypic data, allowing scientists to test antibiotic treatments virtually before conducting human trials [183, 322]. Pathogens such as *E. faecalis*, *N. gonorrhoeae*, and *M. tuberculosis*, which exhibit significant genomic flexibility, can be better understood using these models [323]. The drug development pipeline is accelerated, and the failure rate of candidate antibiotics in later trial phases is reduced using this approach. Phase I investigations assess the safety, tolerability, and pharmacokinetics of healthy volunteers before initiating clinical trials [324]. One example is the effectiveness of delafloxacin and ceftolozane–tazobactam against MDR Gram‐positive and Gram‐negative infections, as demonstrated in their extensive Phase I and II investigations [325, 326]. Phase III trials confirm the performance of an antibiotic under clinical conditions by testing its efficacy in a larger patient population. Cefiderocol was licensed for the treatment of complex urinary tract infections (UTIs) and nosocomial pneumonia caused by bacteria resistant to other antibiotics. In contrast, delafloxacin was later approved for treating severe bacterial skin infections [327].

Aside from infections in the bloodstream and the respiratory tract, one of the most prevalent clinical contexts for antibiotic resistance is extracavity infections, such as UTIs. Complications of UTIs can be caused by bacteria that are resistant to many drugs. These bacteria include *K. pneumoniae* and *E. faecium*, as well as those resistant to fluoroquinolones [328]. Resistance genes are being selected and propagated in both community and hospital settings due to the high frequency of antibiotics used for UTIs on an as‐needed basis. Incorporating extracavity infections into monitoring systems and creating innovative treatment options are crucial, as UTIs are a significant source of antibiotic resistance [329]. At the same time, improvements to delivery are being pursued in preclinical research. There has been evidence of enhanced penetration and prolonged release of antibiotics using nanocarrier systems, including polymeric nanoparticles, metal–organic frameworks, and liposomes. These carriers are beneficial for treating stubborn infections such as drug‐resistant Mycobacterium‐mediated lung disease or Staphylococcus‐induced osteomyelitis [41, 317]. Animal experiments have demonstrated that these platforms enhance bioavailability and mitigate toxicity, particularly useful for treatments with narrow therapeutic windows. Finally, preclinical antibiotic research has been transformed by combining computer techniques, new therapeutics, and conventional animal models. Addressing AMR in various pathogens—including Gram‐negative, Gram‐positive, and atypical bacteria—requires a bridge between preclinical assessments and clinical trials. A multipronged approach that includes phage therapy, AMPs, nanocarriers, and modeling offers a promising foundation for the next generation of antimicrobial research. Table 2 summarizes current results from clinical and preclinical studies, highlighting the importance of the translational pipeline in developing effective treatments to combat bacterial resistance.

**TABLE 2:** An overview of clinical trials and preclinical animal experiments examining the mechanisms of antibiotic resistance in different bacterial species.

Bacterial species	Antibiotic resistance mechanism	Preclinical animal experiments	Clinical trials	References
Carbapenem‐resistant *Enterobacteriaceae* (CRE)	Carbapenemase production (e.g., KPC, NDM, VIM, OXA‐48), efflux pumps	Mouse models were tested for the combination of medications (colistin and carbapenems) as well as phage treatment. Preclinical studies have demonstrated that specific combinations may be effective in overcoming CRE infections.	In Phase III investigations, ceftazidime–avibactam and meropenem–vaborbactam were efficacious against CRE. Cefiderocol and phage therapies are now in Phase II/III clinical trials.	[330, 331]
Multidrug‐resistant *P. aeruginosa*	Efflux pumps, porin mutations, β‐lactamase production	Mouse models have shown that β‐lactamase inhibitors (e.g., ceftolozane–tazobactam) and biofilm‐targeting therapies are effective in treating multidrug‐resistant *P. aeruginosa*.	Ceftolozane–tazobactam and ceftazidime–avibactam have shown potential in Phase III trials for multidrug‐resistant *P. aeruginosa* infections.	[332]
Extended‐spectrum β‐lactamase (EBL)‐producing *E. coli*	ESBL enzyme production (e.g., CTX‐M, TEM, SHV)	Mouse models were used to test the effectiveness of β‐lactamase inhibitors, such as avibactam, when combined with cephalosporins, in combating resistance. Phage therapy is also being studied.	Ceftolozane–tazobactam and ceftazidime–avibactam were shown in clinical trials to be very successful in the treatment of ESBL‐producing *E. coli* infections.	[330]
Carbapenem‐resistant A. *baumannii*	OXA‐type carbapenemase production and efflux pumps	Animal models were used to study phage treatments and β‐lactamase inhibitors, including sulbactam–durlobactam. These medicines have shown potential in overcoming carbapenem resistance in *A. baumannii*.	Clinical studies were successful in treating carbapenem‐resistant *A. baumannii* infections with sulbactam–durlobactam and cefiderocol. Studies on phage treatment are underway.	[333]
Multidrug‐resistant K*. pneumoniae*	Carbapenemase (KPC, NDM) and ESBL production, porin mutations	Combination therapy using colistin, aminoglycosides, and carbapenems was tried in mouse models. Phage therapy and bacteriocin treatments were tested for their capacity to manage resistance.	Ceftazidime–avibactam and meropenem–vaborbactam were evaluated in Phase III studies against carbapenem‐resistant strains. Bacteriocin and phage treatment studies are currently ongoing.	[334]
Multidrug‐resistant *H. pylori*	Mutations in 23S rRNA (clarithromycin resistance), mutations in *gyrA* (fluoroquinolone resistance), and efflux pumps	The mechanisms of clarithromycin and metronidazole resistance were investigated using animal models, including gerbils and mice. These models also examined combination therapies involving new antibiotics and probiotics.	Clinical trials investigated innovative combination therapies for clarithromycin and metronidazole resistance, such as bismuth quadruple therapy. Phage and probiotic research continues.	[335]
Multidrug‐resistant N. *gonorrhoeae*	Mutations in the *penA*, *porB*, and *mtrR* genes (resistance to β‐lactams, fluoroquinolones, and macrolides)	To treat multidrug‐resistant N. gonorrhoeae, mouse models were evaluated with novel antimicrobial peptides and β‐lactamase inhibitors. Probiotic therapy tests try to restore sensitivity in resistant microorganisms.	Phase III studies looked at the effectiveness of ceftriaxone–azithromycin combinations. Novel drugs, such as zoliflodacin and gepotidacin, are now being studied in clinical trials.	[336]
Multidrug‐resistant *Salmonella* spp.	Chromosomal mutations (e.g., *gyrA*), plasmid‐mediated resistance (e.g., *blaCTX‐M*, *QNR* genes)	To treat multidrug‐resistant Salmonella, mice models were studied using novel β‐lactamase inhibitors, efflux pump inhibitors, and phage therapy. The combination therapy with azithromycin was also examined.	New β‐lactamase inhibitors are being tested in clinical trials to establish their efficacy. Trials with oral azithromycin combinations have had variable results.	[337]
Multidrug‐resistant M*. tuberculosis*	Mutations in *katG* (isoniazid resistance), *rpoB* (rifampicin resistance), *embB* (ethambutol resistance), *inhA*, and efflux pumps	Combining medicines like bedaquiline and Delaware in guinea pig and mouse models yielded encouraging results for overcoming resistance. Additionally, immunomodulatory treatments aimed at enhancing the immune response were investigated.	Clinical studies of novel TB drugs such as bedaquiline, delamanid, and pretomanid have shown encouraging results against multidrug‐resistant tuberculosis. Vaccine studies to overcome resistance are still in progress.	[338]
Methicillin‐resistant S*. aureus*	β‐Lactamase production, modified PBP (penicillin‐binding proteins)	Novel β‐lactamase inhibitors were tested in animal models with β‐lactams. Phage therapy has been evaluated for reducing MRSA infections and is successful in decreasing the bacterial load.	Ceftaroline (fifth‐generation cephalosporin) has potential in Phase III tests against MRSA infections. There are ongoing studies investigating alternatives to vancomycin and phage therapies.	[339, 340]
Vancomycin‐resistant *Enterococcus*	Altered target site via *VanA* gene cluster, modifying d‐Ala–d‐Ala to d‐Ala–d‐Lac	To overcome vancomycin‐resistant *Enterococcus* (VRE) resistance, mice models were tested with daptomycin, linezolid, and beta‐lactam combinations. Animal experiments have shown the efficacy of daptomycin in conjunction with β‐lactams.	The trials for daptomycin and linezolid were satisfactory. Novel medications, such as oritavancin and tedizolid, are now being investigated.	[341, 342]

## 6 Challenges, Potential Solutions, and Future Directions

### 6.1 Challenges

**FIGURE 4:**
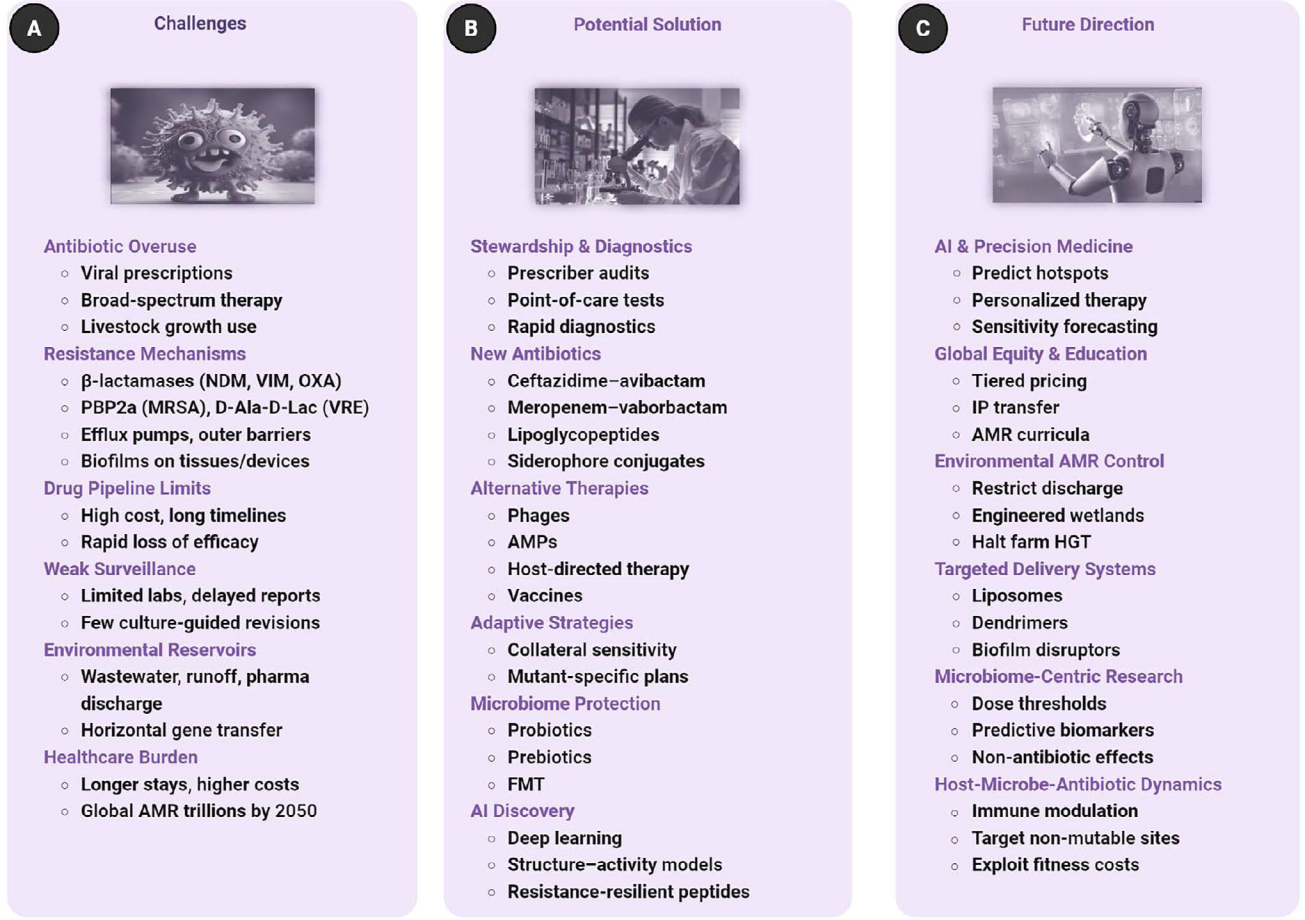
Overview of the problems with antibiotic resistance, possible solutions, and where we might go from here in the future. (A) Obstacles include the widespread use of antibiotics, the diverse range of resistance mechanisms, and the slow pace of drug discovery. (B) Possible answers include bacteriophages, antimicrobial peptides, public stewardship programs, alternative treatments, and rapid diagnostic technologies (such as PCR and CRISPR). (C) Looking ahead: Initiatives to facilitate international collaboration, such as the World Health Organization's global action plan to reduce antibiotic use in agriculture, as well as new drug pipelines and precision medicine utilizing gene therapy and biotherapy, are all on the horizon. Figure created using BioRender.

The emergence of antibiotic‐resistant bacteria is a significant concern for global public health (Figure 4A). Many antibiotics are no longer effective against these microbes due to their varied and complex resistance mechanisms, which have a multiplicative effect on the difficulty of treating infections and the strain on healthcare systems worldwide [343, 344]. While Gram‐negative bacteria such as carbapenem‐resistant *K. pneumoniae*, *P. aeruginosa*, and *A. baumannii* are frequently highlighted due to their formidable resistance profiles, Gram‐positive pathogens like MRSA, VRE, and MDR S. *pneumoniae* also present significant clinical and economic challenges [4, 47, 345]. According to monitoring studies, the isolation of Gram‐positive bacteria, particularly MRSA and *S. aureus* (not *Staphylococcus agalactiae*), has increased in clinical settings, particularly among hospitalized patients.

These microbes can withstand immunological and pharmacological stresses by using various molecular strategies, including enzyme synthesis, target alteration, efflux pumps, and biofilm formation. The β‐lactamase enzymes responsible for breaking down the β‐lactam ring structure of essential antibiotics and rendering them ineffective are found in Gram‐negative pathogens [317]. These enzymes include metallo‐β‐lactamases (NDM, VIM, and IMP), ESBLs, and OXA‐type carbapenemases [8, 47]. On the other hand, MRSA and other Gram‐positive bacteria produce modified PBPs, such as PBP2a, which are resistant to β‐lactams and have a poor affinity for them. Additionally, VRE strains become resistant to vancomycin by modifying the d‐Ala–d‐Ala end of peptidoglycan precursors to d‐Ala–d‐Lac [346, 347]. Other resistance mechanisms in *Staphylococcus* and *Enterococcus* spp. include methyltransferases (cfr) and ribosomal protection proteins (optrA, poxtA), which give both species resistance to oxazolidinones and phenicols. Resistance features are rapidly disseminated across bacterial populations through molecular adaptations, HGT, and spontaneous chromosomal mutations [348]. As a result, infections that were formerly treatable become therapeutic dead ends.

Gram‐positive and Gram‐negative MDR organisms are significant causes of healthcare‐associated infections, especially in intensive care units [235]. Despite the notoriety of CRKP and A*. baumannii* in nosocomial settings, MRSA continues to be a top cause of bacteremia and infections at surgical sites worldwide. Research conducted at tertiary care institutions has shown that the prevalence of *E. faecium* bacteria resistant to β‐lactams and fluoroquinolones is on the rise [349]. In just 10 years, the percentage of penicillin‐resistant bacteria has jumped from half to more than 90%. Given the proliferation of these pathogens, there is an urgent need for innovative approaches to infection management, treatment development, and vaccine research [350, 351]. It is crucial to address Gram‐negative and Gram‐positive threats and challenges as soon as possible, according to the WHO's Bacterial Priority Pathogens List for 2024, [352, 353]. Due to the clinical risks posed by Gram‐positive and Gram‐negative bacteria, including MRSA and VRE, *S. pneumoniae* is classified as a medium‐priority organism, whereas *S. aureus* is regarded as a high‐priority organism [354].

The costly and time‐consuming procedure of creating new antimicrobial drugs is one of the most enduring obstacles to addressing AMR. The time and money needed to bring a novel antibiotic to market can easily surpass $1 billion, and the process can take more than a decade [355]. Pharmaceutical companies have few financial incentives to develop new treatments, despite the growing need for such treatments. Gram‐negative bacteria have more intricate chemical structures that require them to cross their outer membrane and evade resistance enzymes, which is particularly true for medicines that target these bacteria [356]. Yet, Gram‐positive infections, such as MRSA and VRE, are also changing at a rapid pace, and they frequently develop resistance to newly administered medications within a few years of their deployment [357]. Consider the growing evidence linking linezolid resistance in VRE and MRSA to transferable plasmids. This raises concerns about the future effectiveness of last‐line treatments such as tedizolid. Regulatory obstacles, uncertain market returns, and inadequate payment models contribute to a lack of investment in antibiotic innovation, which hinders antibiotic research [358, 359].

Antibiotic overuse and improper administration in human and animal medicine accelerate antibiotic resistance. It is common practice to prescribe antibiotics in clinical situations without proper evidence of the disease, such as when treating viral infections for which antibiotics have no effect or when the pathogen has not been confirmed through culture‐based testing [360]. This technique is common in all countries, regardless of income level, and it plays a significant role in selecting resistant strains [285, 355]. In addition, empirical therapy, including broad‐spectrum antibiotics, unknowingly promotes resistance in both commensal and pathogenic bacteria. Antibiotics are commonly administered to cattle in agriculture for both medical and growth‐promoting purposes. This practice leads to the evolution of resistant zoonotic bacteria, such as Salmonella, Campylobacter, and Enterococcus, which can be transmitted to humans through food or direct contact [355, 361]. There is an unacknowledged strain on microbial ecosystems resulting from the discharge of antibiotics into the environment by pharmaceutical companies and agricultural runoff. This strain helps bacteria in the environment develop resistance genes, which can then be passed on to humans [362].

Another obstacle to AMR management is the absence of a cohesive worldwide strategy. Many areas, particularly in LMICs, lack the infrastructure, monitoring systems, and quick diagnostic tools necessary to execute effective stewardship programs, even though several nations have established national action plans [363, 364]. Overuse of broad‐spectrum drugs in an ad hoc manner promotes resistance development and delays proper therapy due to the lack of quick diagnoses [20, 333]. Further complicating AMR control in these settings are shortages of qualified workers, inadequate laboratory capacity, and limited access to quality‐assured drugs [365]. According to retrospective research, the fact that only a small percentage of empirical antibiotic prescriptions are modified after culture at certain tertiary hospitals suggests serious deficiencies in diagnosis. Another obstacle to eradicating infections is the fundamental biological and structural characteristics of bacteria. The outer membrane of Gram‐negative bacteria forms a permeability barrier that prevents antibiotics from penetrating the cell, and efflux pumps actively expel the antibiotic treatments. Biofilm development and thick peptidoglycan coatings are two additional mechanisms by which Gram‐positive bacteria evade drugs and immune effectors. Forming biofilms on tissue and medical equipment surfaces can make microbial communities up to a thousand times more resistant than those in water. The biofilm‐producing strains of *S. aureus*, *P. aeruginosa*, and others are commonly associated with persistent infections, such as endocarditis, ventilator‐associated pneumonia, and chronic wounds [333, 366]. Aside from blocking the transport of antibiotics, the biofilm matrix allows for HGT, which speeds up evolution and maintains resistance features in their current location. This makes treatment more challenging and necessitates more intensive and time‐consuming methods, which are not always effective.

The financial toll of AMR is likewise high. Healthcare systems are overwhelmed as a result of infections produced by resistant bacteria, which extend hospital stays, raise mortality and morbidity rates, and necessitate the use of last‐line or experimental therapies more frequently. The lack of modern diagnostic tools, treatment options, and robust infrastructure in LMICs makes them more vulnerable [355]. Healthcare costs and hospital resources are strained in even the most industrialized nations due to increased resistance and incorrect prescribing practices [367]. Additional justification for the necessity of concerted worldwide action is provided by projections that the global economic effect of AMR, if left unchecked, might reach trillions of dollars by 2050. Innovation in science is essential, but so is political will, public health education, international collaboration, and the resolution of these complex problems. To reduce the spread of resistance, it is vital to implement region‐specific stewardship programs, increase investment in rapid diagnostics, and support antibiotic research more heavily. A complete understanding of resistance dynamics and the ability to execute more focused therapies are enhanced when clinical research and surveillance programs include both Gram‐positive and Gram‐negative organisms [4].

In conclusion, AMR is a complex and ever‐evolving problem that affects nearly every type of bacterium. The MDR phenotypes and difficult‐to‐penetrate membranes of Gram‐negative species receive the most attention. Still, Gram‐positive bacteria, such as MRSA, VRE, and *S. pneumoniae*, also pose a danger due to their unique resistance mechanisms. Over the last decade, mounting evidence has shown that Gram‐positive pathogens are becoming increasingly resistant to antibiotics; this trend warrants the same level of attention from researchers and policymakers as that directed toward Gram‐negative bacteria. The pressing need for new antibiotics, improved diagnostics, education on appropriate antibiotic use, and increased international cooperation to protect public health is heightened as biological, economic, and policy‐related issues converge.

### 6.2 Potential Solutions and Future Directions

A multifaceted strategy that includes not only treating infections but also anticipating, preventing, and responding to the emergence of resistant strains of Gram‐negative and Gram‐positive bacteria is necessary to combat antibiotic resistance effectively [355] (Figure 4B,C). Scientific efforts must expand to include a broader range of microbial threats and host conditions, accounting for the fact that resistance mechanisms differ in complexity and effect across bacterial species [147]. The primary focus should be on enhancing global monitoring networks and surveillance systems to track antibiotic usage and changes in resistance. These systems must detect new resistance phenotypes in bacteria, utilizing predictive analytics and real‐time data collection. There are both established dangers, such as Gram‐negative bacteria that are resistant to carbapenems (e.g., *K. pneumoniae* and *P. aeruginosa*), and newer, more complex risks, including Gram‐positive bacteria that are resistant to methicillin and vancomycin, as well [368, 369]. Researchers and policymakers can use a global platform that integrates molecular diagnostics, clinical outcomes, and epidemiological trends to respond proactively to AMR hotspots [370].

The fight against AMR relies heavily on pharmacological innovation. By blocking key carbapenemase enzymes, new antibiotic combinations have been developed, which have restored efficacy against Gram‐negative infections resistant to multiple drugs [371]. Examples of these combinations are ceftazidime–avibactam and meropenem–vaborbactam. Similarly, the arsenal against Gram‐positive infections, such as MRSA and resistant Streptococci, has been augmented by lipoglycopeptides like dalbavancin and oritavancin [372, 373]. In addition to increasing therapy options, these medicines may serve as a proof‐of‐concept for developing molecules with improved pharmacokinetics and reduced potential to drive resistance [374]. A significant improvement in the ability to combat Gram‐positive infections has been the development of MAAs [375]. According to Jia et al. [19], a new class of antibiotics called MAAs was created by structurally engineering molecules to have three or four arms derived from phenylbenzoic acid and a core unit, such as ethylene, benzene, triazine, carbon, or nitrogen. Inhibiting lipid carrier molecules essential for cell wall formation, the combination of the core and arms exclusively targets Gram‐positive bacteria, even though neither component has antibacterial action. Regarding MRSA and other clinically significant isolates of Gram‐positive bacteria, these MAAs exhibit significant activity [376, 377]. This novel approach exemplifies a new path in antibiotic research by facilitating the scaffold‐based creation of selective, effective, and potentially less prone to resistance compounds.

In addition, siderophore‐conjugated drugs, such as cefiderocol, have expanded the realm of antibiotic design. Cefiderocol can achieve active absorption by targeting the bacterial iron transport mechanism [375]. This is especially true in Gram‐negative bacteria, which often possess very robust outer membrane barriers or numerous efflux systems. Researchers are investigating the use of synthetic carriers, such as dendrimers and liposomes, in targeted delivery systems for Gram‐positive infections to enhance antibiotic penetration into the biofilm matrix and host tissues [378]. Additionally, reassessing and improving current antibiotic classes to treat long‐lasting Gram‐positive infections is becoming increasingly critical [12]. In situations such as endocarditis and prosthetic joint infections, agents like glycopeptides, oxazolidinones, and newer lipopeptides like daptomycin have shown efficacy in the long‐term treatment of MRSA and VRE infections [374, 376]. Therapeutic drug monitoring and tailored pharmacokinetic modeling are crucial for treatment efficacy in these long‐term diseases. To optimize effectiveness while minimizing toxicity and risk of resistance, these techniques enable dosage modification based on individual patient factors.

To account for the diversity in healthcare systems worldwide, antimicrobial stewardship must evolve in tandem with the development of new treatments and therapies. Outpatient settings, veterinary clinics, and agricultural enterprises should all be part of stewardship initiatives, not only tertiary institutions. Appropriate antibiotic selection, dose, and duration should be ensured across all sectors by tailored interventions such as prescriber audits, decision‐support tools, and point‐of‐care diagnostics. Crucially, these programs must include severe Gram‐negative sepsis and high‐burden Gram‐positive infections, such as those in soft tissues and skin [379]. Recent technological developments in diagnostic techniques have greatly enhanced the ability to quickly and accurately identify resistance genes. Rapidly detecting resistance determinants, such as *bla_KPC*, *mecA*, *vanA*, or *erm* genes, is possible using platforms like NGS, CRISPR‐based diagnostics, or multiplex PCR [380]. When combined with electronic health records and analytics powered by AI, these technologies can potentially steer patients toward more personalized treatment plans and curb the overuse of antibiotics.

Additionally, a strong paradigm in AMR control using collateral sensitivity has been proposed [381]. This study demonstrated that the development of resistance can be suppressed in vitro by strategically matching drugs and identifying bacterial mutants that exhibit hypersensitivity to one antibiotic in response to resistance to another. An idea known as “mutant‐specific collateral sensitivity” enables the development of adaptive treatment plans that leverage resistance trade‐offs to restrict evolutionary escape mechanisms [382]. Nontraditional antimicrobial methods are gaining traction rapidly as supplementary or alternative measures to conventional antibiotics. Even infections caused by Gram‐negative (e.g., *A. baumannii*) and Gram‐positive (e.g., *S. aureus*) bacteria can be effectively treated using bacteriophage therapy, which employs viruses that are unique to the host to target and destroy bacterial cells [375]. Phage resistance may be dynamically countered by their ability to evolve in response to bacterial targets.

Nevertheless, large‐scale clinical studies are necessary to evaluate these constraints, including immune clearance, limited host range, and regulatory barriers. Pexiganan and LL‐37 are synthetic analogues of AMPs, representing another promising area for reducing AMR. AMPs can interfere with bacterial communication, disturb membrane integrity, and decrease nucleic acid synthesis, exhibiting broad‐spectrum action. They show promise as candidates for topical or systemic use due to their mechanisms’ reduced susceptibility to resistance development [383, 384]. Furthermore, peptide engineering and nanotechnology are developing new techniques to enhance ampicillin's stability, bioavailability, and selectivity [385, 386].

Instead of targeting infections directly, host‐directed treatments aim to enhance the host's immune system. These treatments aim to improve bacterial clearance while minimizing tissue damage by regulating immunological signaling, autophagy, and metabolic pathways [369, 387]. New therapeutic targets, such as de novo purine biosynthesis, provide hope beyond the current arsenal of anti‐TB treatments. A first‐in‐class inhibitor targeting mycobacterial PurF—the gateway enzyme in purine biosynthesis—was recently found by Lamprecht et al. [388]. Genetic confirmation and single‐cell microscopy demonstrated that this small molecule specifically disrupted DNA replication, resulting in nanomolar bactericidal action against TB. The study's most important finding is that nucleobase concentrations in human lungs are too low to use salvage pathways to circumvent PurF suppression. As a promising option for next‐generation TB therapy, JNJ‐6640 synergized with current regimens to combat drug‐resistant bacteria, and its long‐acting injectable formulation demonstrated substantial in vivo effectiveness. Interferons, checkpoint inhibitors, metabolic reprogrammers, and other immune modulators have shown potential in the adjuvant treatment of TB and MRSA‐related sepsis, two of the most challenging infectious diseases to manage. In the long run, vaccination remains one of the most effective ways to reduce AMR [389, 390]. Not only can vaccines prevent infections from happening, but they also lessen the need for antibiotics, which in turn slows the development of resistance. For instance, pneumococcal conjugate vaccines have decreased antibiotic use and invasive infections caused by *S. pneumoniae* [391]. Similarly, vaccines against Gram‐negative bacteria, such as *K. pneumoniae* and *P. aeruginosa*, are currently under development; however, obstacles remain due to the variety of their antigenic components and their ability to evade the immune system [392]. In recent years, there has been a surge in interest in the microbiome and its potential involvement in AMR. It is common for antibiotic‐resistant bacteria to spread when they disrupt microbial ecosystems [393].

In addition to antibiotics, new research indicates that treatments that do not include antibiotics can significantly impact the ability of enteropathogens to colonize. Necessary research by Grießhammer et al. [394] found that 28% of 53 pharmaceuticals, including antihistamines, antipsychotics, and calcium channel blockers, promoted the growth of Salmonella Typhimurium and other enteropathogens in the gut communities. These nonantibiotics worked by selectively inhibiting commensal bacteria and increasing metabolic flexibility in pathogens, thereby allowing enteropathogens to exploit previously occupied nutritional niches. Terfenadine and similar medicines compromised microbiota‐mediated colonization resistance in mouse models, resulting in a 10‐ to 100‐fold increase in intestinal pathogen burdens, accelerated disease onset, and exacerbated inflammation. Previously unrecognized risk factors for enteric infections are now being identified through this approach.

Probiotics, prebiotics, and fecal microbiota transplantation have all shown promise in restoring microbiome balance and warding against recurring infections, especially those caused by *C. difficile*. Reducing colonization by MDR organisms in the gut and skin, as well as modifying the microbiome, also provides prophylactic therapy for immunocompromised individuals. By optimizing antibiotic usage, anticipating the development of resistance, and accelerating drug discovery, AI has become a game‐changing tool in the fight against AMR. AI systems, such as deep learning and machine learning algorithms, can sift through massive chemical libraries in search of probable therapeutic candidates with promising molecular features and antibacterial activity [395, 396]. For example, novel antibiotics active against *E. coli* and *K. pneumoniae* have been discovered by screening over 6,000 compounds using AI models [396, 397].

Additionally, AI enables structure–activity relationship modeling, which helps predict how new compounds will interact with bacterial targets and withstand potential mutations [398]. AI has produced AMPs to enhance therapeutic indices [399, 400] by predicting peptide–membrane interactions and optimizing peptide stability, potency, and specificity. Finding molecules with improved pharmacological profiles is guided by deep learning methods that mimic drug–pathogen interactions [401, 402]. Next‐generation medicines with built‐in resilience against bacterial adaptation can be designed using AI, which identifies structural motifs that are resilient to resistance mutations [395, 398].

AI‐integrated decision‐support systems optimize antibiotic prescriptions in clinical settings by integrating pathogen profiles with patient‐specific data, thereby reducing the use of empirical antibiotics. This means that AI does more than speed up the discovery process; it also helps to close the gap between what happens in the laboratory and what patients experience. Future AMR treatments may be built upon an integrated AI strategy, which supplements established approaches, including bacteriophage therapy, AMPs, host‐directed therapies, vaccinations, and microbiome interventions [403, 404]. Identifying the root causes of environmental factors that contribute to AMR is equally essential. There are drugs, bacteria, and genes for antibiotic resistance in wastewater from healthcare facilities, pharmaceutical factories, and cattle ranches. These contaminants find their way into ecosystems where they promote bacterial HGT. To reduce ecological AMR reservoirs, policy frameworks should mandate more stringent limits for antimicrobial discharge and increase the scale of bioremediation methods such as engineered wetlands and enzymatic degradation.

Education and awareness campaigns are crucial in the fight against antimicrobial resistance. To address antibiotic misuse in homes, healthcare institutions, and the animal husbandry business, initiatives that modify behavior based on evidence and cultural sensitivity are necessary. Incorporating AMR ideas into the training curriculum may help ensure that medical, veterinary, and pharmacy students begin their careers by learning to prescribe antibiotics safely. Social media and community health platforms can also help disseminate information about proper infection prevention and antibiotic stewardship practices. Global equity is essential in the fight against antibiotic resistance. Many countries with low to medium economic levels face challenges in accessing diagnostics, the quality of medicines, and inadequate regulation in the antibiotic market. To ensure a fair distribution of innovative diagnostics, vaccines, and antibiotic therapies, multinational collaborations should prioritize the transfer of intellectual property, implement tiered pricing, and consolidate procurement processes. Raising global financing to support AMR research in low‐resource countries is vital so that everyone, not just those in wealthy nations, may benefit from it.

The resolution of fundamental scientific puzzles, particularly those revealed by new information regarding the risks of chemicals other than antibiotics, should be the ultimate objective of AMR management, notwithstanding the hopeful outcomes of multipronged methods. Overcoming the varied problem of resistance development requires answering the complex and critical scientific concerns about next‐generation antimicrobial therapies. An essential tactic is to develop antibiotics or combinatorial regimens such as teixobactin or MAAs, that target nonmutable sites or employ collateral sensitivity networks, thereby compelling evolution to make trade‐offs. The ultimate objective of these methods is to render resistance functionally unsustainable by limiting the adaptability of bacteria through fitness costs or incompatible mutational pathways—a structure for predicting expected resistance also accompanies this. Combining data on pharmaco–microbiome interactions with host physiological parameters and real‐time genomic surveillance presents a substantial opportunity for AI systems to anticipate the establishment of antibiotic and nonantibiotic drug resistance. Permeability barriers in Gram‐negative bacteria remain a significant obstacle.

To circumvent these natural defenses without unintentionally selecting for mutations in efflux or porin, scientists need to create new methods, including lipid‐based vesicles, membrane‐penetrating nanocarriers, and siderophore–drug conjugates. Avoiding these problems without triggering compensatory resistance mechanisms is a primary goal of the design process. In addition, biofilm eradication efforts, particularly for long‐term Gram‐positive infections, require therapies that can penetrate the biofilm matrix to sterilizing concentrations. To achieve this goal, drugs that disrupt biofilm structure or activate dormant cells can be used in conjunction with targeted delivery systems, such as liposomes or dendrimers. One uncharted area in the investigation of antibiotic efficacy against intracellular infections might be the host–microbe–antibiotic dynamics, which could be addressed by host‐directed therapy. Safe manipulation of immunological mechanisms, such as autophagy, macrophage polarization, and inflammasome activity, can enhance antimicrobial efficacy without harming beneficial microorganisms. We urgently need a standardized framework for antibiotic–adjuvant combinations to simplify treatment regimens.

By integrating data on pharmacokinetics and pharmacodynamics with data on evolutionary constraints, this model aims to forecast which drug combinations will initially prevent compensatory mutations from happening. Learning how the microbiota may bounce back is also critical. It is possible that certain nonantibiotic drugs, such as proton pump inhibitors, terfenadine, and metformin, may enhance the metabolic flexibility of infections through specific biochemical pathways while selectively inhibiting commensal bacteria. Upon consideration, alterations in antibiotic resistance and host‐mediated factors, including immunological signaling and mucosal integrity, facilitate the propagation of infection. To address environmental resistance from a more comprehensive ecological viewpoint, measures must be taken to halt the HGT and the selection of resistance elements generated by antibiotic and nonantibiotic pressures in wastewater, farms, and industries.

Last, determining drug‐microbiome dose–response thresholds is critical for preventing microbiome collapse. It is a crucial but challenging endeavor to resolve nonantibiotic doses that significantly reduce colonization resistance in various human populations. By developing predictive biomarkers, it may be possible to avert drug‐induced overgrowth of enteropathogens. Changes in microbial metabolic capacity or signs of significant commensal depletion are two examples of potential indications. This might pave the way for early intervention and risk categorization. Integration at the systems level, spanning fields such as molecular microbiology, clinical pharmacology, ecology, and AI, is crucial to address these complex research difficulties and drive the next generation of antimicrobial innovation.

## 7 Conclusion

Antibiotic resistance affects both Gram‐positive and Gram‐negative bacteria, and is becoming an increasingly serious global health issue. Even though bacteria like *K. pneumoniae*, *P. aeruginosa*, and *A. baumannii* have been studied for their complex resistance traits like β‐lactamase production, efflux pump activation, and reduced membrane permeability, Gram‐positive pathogens like *S. aureus*, *E. faecium*, and *S. pneumoniae* also present serious clinical problems due to mechanisms such as altered PBPs, modified target sites, and vancomycin resistance. These pathogens impose significant socioeconomic costs globally, collectively responsible for an increasing number of diseases that are difficult to cure, longer hospital admissions, and higher mortality rates.

Multiple mechanisms, including genetic mutations, biofilm formation, metabolic rewiring, enzymatic inactivation, and HGT, contribute to the development and dissemination of resistance. Bacteria can evade current treatments and survive in hostile environments due to these complex mechanisms, which arise across various environmental, agricultural, and clinical contexts. To develop targeted, effective, and long‐term treatments, it is crucial to gain a deeper understanding of these underlying processes. This review underscores the critical need to do so for all bacterial pathogens, not just Gram‐negative ones. A global approach that combines innovation with execution is necessary to combat AMR. Encouraging options are under active investigation, such as bacteriophage‐based therapies, AMPs, CRISPR‐based gene‐editing technologies, and next‐generation antibiotics.

Genome‐based resistance profiling and rapid molecular diagnostics must be improved and integrated to enable the prompt administration of therapy. Global partnerships that will enhance antimicrobial stewardship, public health education, and access to medical breakthroughs for everyone are equally crucial. For a coordinated and successful response, low‐ and middle‐income nations, often on the front lines of AMR, require financial backing, improved infrastructure, and the transfer of relevant technologies.

In summary, antibiotic resistance is an ever‐evolving, complex problem with no bounds in terms of bacterial taxonomy or geography. Science must continue to innovate, international collaboration must be strong, and knowledge must be strategically translated into action if antimicrobial agents are to remain effective for centuries to come. The world can combat the rising threat of antibiotic resistance and ensure a healthier future by combining efforts in microbiology, medicine, policy, and public involvement.

## Author Contributions

Conceptualization: R.G.E., M.B., Z.L., A.H.S., and Z.Z. Writing—original draft preparation: R.G.E. Writing—review and editing: R.G.E., A.H.S., Y.D., M.B., and Z.Z. Supervision: Z.Z. Funding: Z.Z. Project administration: Z.Z. All authors have read and approved the final manuscript.

## Conflicts of Interest

The authors declare no conflicts of interest.

## Ethics Statement

The authors have nothing to report.

## Data Availability

The authors have nothing to report.
